# Short-chain fatty acids abrogate Japanese encephalitis virus-induced inflammation in microglial cells via miR-200a-3p/ZBTB20/IKβα axis

**DOI:** 10.1128/mbio.01321-24

**Published:** 2024-06-13

**Authors:** Atreye Majumdar, Indira Priya Siva Venkatesh, Vivek Swarup, Anirban Basu

**Affiliations:** 1 National Brain Research Centre, Manesar, Haryana, India; 2 Department of Neurobiology and Behaviour, University of California, Irvine, California, USA; 3 Institute for Memory Impairments and Neurological Disorders (MIND), University of California, Irvine, California, USA; Virginia Tech, Blacksburg, Virginia, USA

**Keywords:** short-chain fatty acids, microglia, microRNA, flavivirus, inflammation

## Abstract

Japanese encephalitis virus (JEV), a member of the Flaviviridae family, is a leading cause of viral encephalitis in humans. Survivors of this infection often develop lifelong neurological sequelae. Short-chain fatty acids (SCFAs) produced in the gut are vital mediators of the gut-brain axis. We aimed to study microRNA-based mechanisms of SCFAs in an *in vitro* model of JEV infection. N9 microglial cells were pretreated with SCFA cocktail before JEV infection. Cytokine bead analysis, immunoblotting, and PCR were performed to analyze relevant inflammatory markers. microRNA sequencing was performed using Illumina Hiseq, and bioinformatics tools were used for differentially expressed (DE) miRNAs and weighted gene co-expression network analysis (WGCNA). microRNA mimic/inhibitor experiments and luciferase assay were performed to study miRNA-target interaction. A significant reduction in monocyte chemoattractant protein (MCP1) and tumor necrosis factor alpha (TNFα) along with reduced expression of phospho-nuclear factor kappa B (phospho-NF-κB) was observed in SCFA conditions. Significant attenuation of histone deacetylase activity and protein expression was recorded. miRNA sequencing revealed 160 DE miRNAs in SCFA + JEV-treated cells at 6 h post-infection. WGCNA revealed miR-200a-3p, a hub miRNA significantly upregulated in SCFA conditions. Transcription factor ZBTB20 was bioinformatically predicted and validated as a gene target for miR-200a-3p. Further miRNA mimic/inhibitor assay demonstrated that miR-200-3p regulated ZBTB20 along with Iκβα that possibly dampened NF-κB signal activation downstream.

**IMPORTANCE:**

The gut-brain axis plays a pivotal role in the physiological state of an organism. Gut microbiota-derived metabolites are known to play a role in brain disorders including neuroviral infections. Short-chain fatty acids (SCFAs) appear to quench inflammatory markers in Japanese encephalitis virus-infected microglial cells *in vitro*. Mechanistically, we demonstrate the interaction between miR-200a-3p and ZBTB20 in regulating the canonical nuclear factor kappa B (NF-κB) signaling pathway via transcriptional regulation of Iκβα. Findings of this study pave the way to a better understanding of SCFA mechanisms that can be used to develop strategies against viral neuroinflammation.

## INTRODUCTION

Japanese encephalitis is an acute neuroinflammatory disease caused by the Japanese encephalitis virus (JEV), a member of the Flaviviridae family (1). JEV is a positive single-stranded RNA virus predominantly reported in tropical and sub-tropical regions geographically. Despite the availability of a vaccine, approximately 70,000 symptomatic cases of JEV are reported annually, causing death in 30% of the patients while 50% of survivors experience permanent neurological damage (2). JEV invades the host through the infected *Culex* mosquito bite and, after creating sufficient peripheral viremia, enters the central nervous system (CNS) (3, 4). Within the brain, JEV targets neuronal cells, astrocytes, microglia, and endothelial cells, causing severe neuroinflammation and neuronal death (5). In search for a strategy to combat JEV-led neuroinflammation, we look toward the gut.

In the past decade, the gut-brain communication has garnered well-deserved attention. Studies involving neuroinflammatory disorders have demonstrated significant changes in host gut microbial taxa (6, 7). One of the key channels of this communication is through metabolites produced by the gut microbiota. Even though the gut microbiota has variability across species, the functional aspect of the microbiome via metabolite function is evolutionarily conserved (8). Short-chain fatty acids (SCFAs), such as acetate, propionate, and butyrate, are small organic metabolites secreted upon the fermentation of complex carbohydrates by the intestinal bacteria, dominantly Firmicutes and Bacteroidetes. Maintained at a stable proportion in healthy states, alterations in SCFA levels have been observed in a spectrum of brain disorders (9). Whether these alterations are the cause or effect or part of a feedback during disease are burning questions in the field. As one of the primary agents mediating the gut-brain axis, the role of SCFAs as immune-modulatory agents has emerged in the literature concerning neurodegenerative diseases (10, 11). Within host cells, SCFAs have been reported to regulate gene expression epigenetically via various mechanisms such as DNA methylation and histone post-translational modifications or by regulating microRNA (miRNA) expression (12). There is scant evidence of the gut-brain axis in context to viral infections. The status of the gut microbial profile of hosts carrying neurotrophic viral infections is still largely unaddressed (6). Hence, exploring the molecular mechanisms of gut-derived SCFAs is a step forward toward understanding the latter’s impact on virus-led neuropathology.

Microglial cells are the most abundant mononuclear phagocytes in the CNS (13, 14). They are capable of immune surveillance, chemotaxis, clearing out pathogens and unwanted synapses, phagocytosis, cytokine production, and antigen presentation (13, 15). Activated microglia are characterized as having an altered proliferation, morphology, and phagocytic activity and releasing a plethora of inflammatory signaling molecules, cytokines, and chemokines. Our previous reports on JEV have established microglia as essential neuroimmune regulators in JEV infection (16–18). Even though SCFAs and microglia interactions have been subject of a few studies (19, 20), we found a lacuna in distinct molecular mechanisms within the host microglia underlying the anti-inflammatory function of SCFAs.

miRNAs are small non-coding RNAs that regulate over 30% of protein coding genes and fine tune fundamental cellular activities (21). Our group has conducted several studies on the role of host microRNA in JEV-mediated neuropathogenesis paving way for a possible therapeutic target. Host miR301a has been reported to block transcription factor (TF) interferon regulatory factor 1 (IRF1), thus bringing down the expression of type 1 interferon (IFN) required for antiviral immune response (22). miR-155, miRNA-432, and miR-451a have also been shown to play critical roles in regulating the JEV-mediated pathogenesis (23–25). Studies have shown the co-regulatory mechanisms between histone deacetylases (HDACs) and microRNA expression changes. There exists a fine balance between the two machineries in the cell (26, 27). HDAC inhibition has been used to target miRNA and other non-coding RNAs in disease conditions (28). Based on these findings, we believe HDAC inhibition using SCFAs is likely to have an effect on regulation of transcripts such as miRNAs, especially in JEV infection where the miRNAs are critically involved. It was our interest to investigate the possible effect of combined acetate, propionate, and butyrate in modulating the host miRNA machinery to defend against JEV-induced mechanisms within the cellular environment.

In this study, we hypothesize that SCFAs regulate miRNA-led gene expression and hence abrogate JEV-induced inflammation in mouse microglial cell model. To prove this, we went on to investigate inflammatory pathways, microRNA expression profile, and HDAC expression changes to obtain mechanistic insights of SCFAs within microglial cells *in vitro*. This study provides evidence of SCFA-induced miR-200a-3p in regulation of the NF-κB pathway, consequently alleviating cyto/chemokine storm produced by an infected host cell.

## RESULTS

### Alleviated cyto/chemokines and phosphorylated NF-κB in JEV-infected cells upon SCFA pretreatment

**Fig 1:**
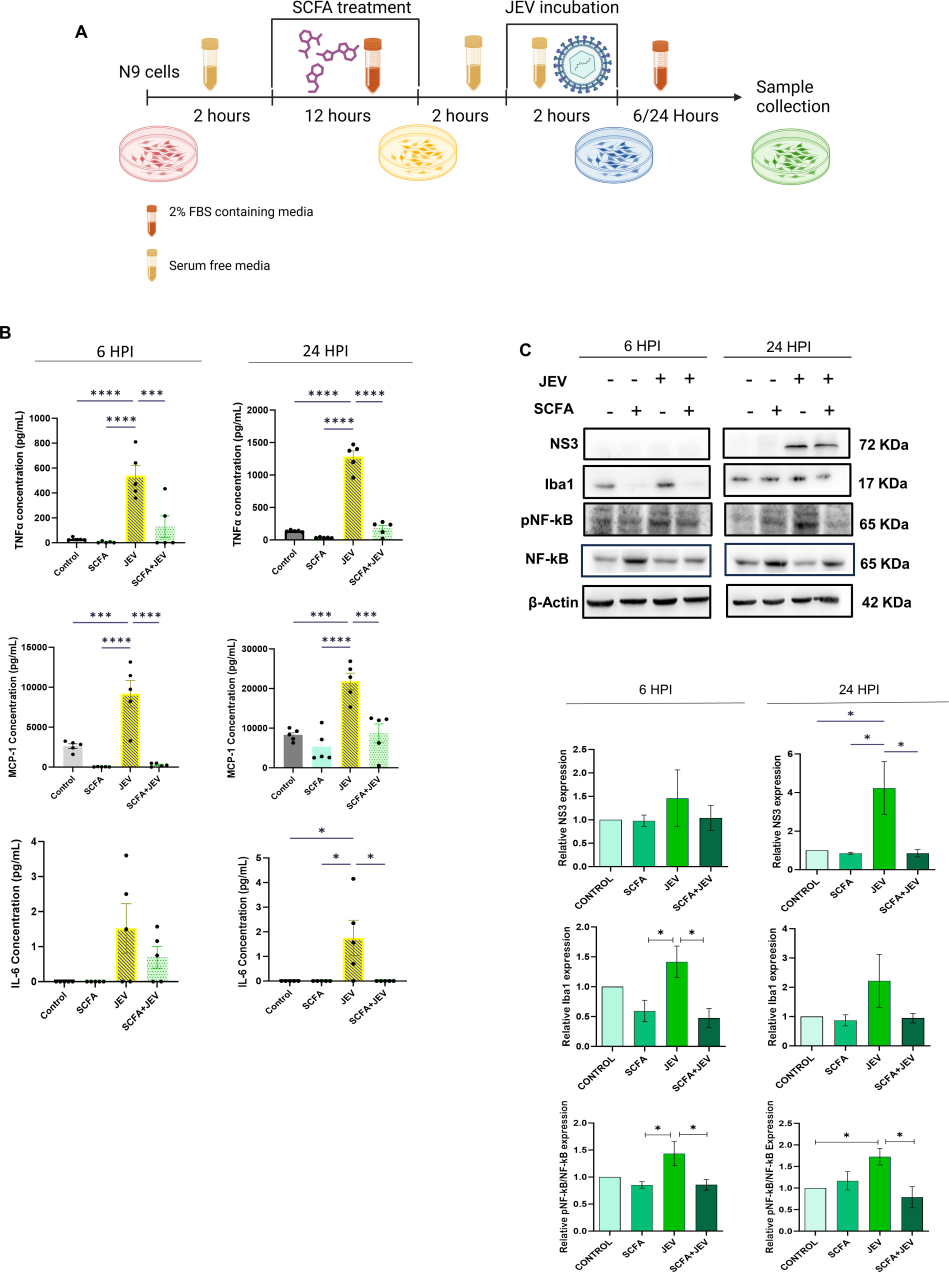
SCFA pretreatment reduces expression of inflammatory markers post-JEV infection in microglial cells. (**A**) Schematic representation of the experimental paradigm followed. (**B**) Cells supernatant isolated form N9 microglial cells post SCFA/PBS pretreatment and/or JEV infection (multiplicity of infection [MOI] 3 ) were subjected to cytokine bead analysis. Graphs on the upper panel (blue dot) represent cytokine expression 6 HPI and those on the lower panel (orange dot) represent 24 HPI. Data presented as absolute concentration (µg/mL) ± SEM. (**C**) Representable immunoblots from cell lysate showing various inflammatory markers at 6 HPI and 24 HPI post SCFA pretreatment and JEV infection. (**D**) Bar plots showing densitometric quantification of the immunoblots in panel B. Data represented as mean fold change ± SEM with respect to control from a minimum of three independent experiments. *P*-values were determined (*, *P* < 0.05; **, *P* < 0.01; ***, *P* < 0.001) using one-way analysis of variance and Tukey’s *post hoc* correction.

Among the six cyto/chemokines detectable by the array, we observed a significant rise in MCP1, TNFα, and interleukin 6 (IL6) in JEV-infected N9 cells at 6 h post-infection (HPI) and 24 HPI (Fig. 1B). This elevated expression was reduced when cells were pretreated with SCFA cocktail comprising acetate, butyrate, and propionate before infection (SCFA + JEV). SCFA pretreatment control (SCFA) did not observe any rise in cyto/chemokines as expected. SCFAs were successful in suppressing a rise in cyto/chemokines in the presence of JEV at both time points. In JEV-infected conditions, we observed a higher expression of MCP1 and TNFα at 24 HPI, while IL6 was relatively lower at 24 HPI. SCFA-led suppression in the SCFA + JEV condition was consistently significant across both time points (Fig. 1B). Immunoblotting revealed a reduction in NS3 viral protein at 24 HPI. Alongside, a decrease in phosphorylated NF-κB (pNF-κB) and Iba11 was observed. pNF-κB was reduced by SCFA pretreatment at both time points. Iba1 reduction was more pronounced at 6 HPI and not significant at the later time point 24 HPI (Fig. 1C and D).

### SCFA administration rescues pan HDAC overexpression caused by JEV

**Fig 2:**
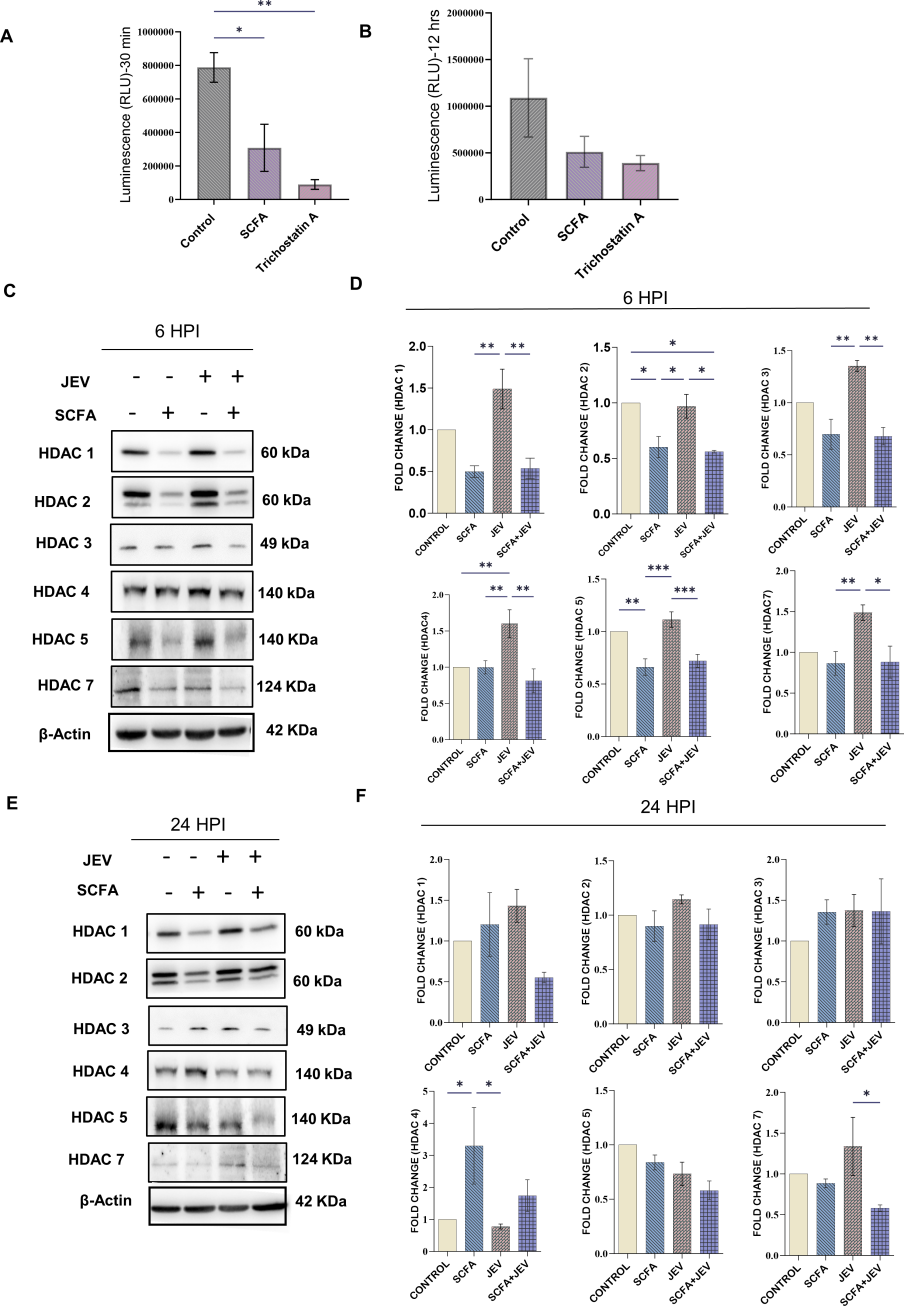
HDAC activity and protein expression analysis post SCFA pretreatment and JEV infection. HDAC activity assay was performed using HDAC Glo I/II kit (Promega) to assess the HDAC inhibiting efficiency of SCFA cocktail. This assay measures luminescence as a marker of functional HDACs within a sample. Loss of luminescence indicates inhibition of HDAC enzymatic activity. (**A**) Represents the relative luminescence units (RLU) measured 30 min post SCFA treatment in N9 cells. (**B**) Represents the relative luminescence units measured 12 h post SCFA treatment in N9 cells. Data are represented as RLU ± SEM with respect to control from a minimum of three independent experiments. Trichostatin A was used as positive control. (**C**) Representable immunoblots of total HDAC protein from whole cell lysate after desired treatment paradigm using SCFA and/or JEV (multipicity of infection [MOI] 3) 6 HPI. (**D**) Bar graphs showing the densitometric quantification of the immunoblots from panel C. (**E**) Representable immunoblots of total HDAC protein from whole cell lysate after desired treatment paradigm using SCFA and/or JEV (3 MOI) 24 HPI. (**F**) Bar graphs showing the densitometric quantification of the immunoblots from panel E. Data represented as mean fold change ± SEM with respect to control from a minimum of three independent experiments. *P*-values were determined (*, *P* < 0.05; **, *P* < 0.01; ***, *P* < 0.001) using one-way analysis of variance and Tukey’s *post hoc* correction.

The SCFA cocktail was assessed for its ability to reduce HDAC class I/II activity. The decline in activity was observed post 30 min of SCFA treatment up to 12 h of the same (Fig. 2A and B). Immunoblotting demonstrated a significant rise in cellular HDACs 1–5 and 7 in JEV-infected conditions. This was mitigated when the cells were pretreated with SCFAs as shown in SCFA and SCFA + JEV conditions. This effect was observed at 6 HPI (Fig. 2C and D). A different pattern was observed at 24 HPI where SCFAs were unable to reduce the HDAC protein expression significantly in HDACs 1, 2, 3, and 5 (Fig. 2E and F). HDAC 4 had an inverse trend at 24 HPI compared to 6 HPI. However, HDAC 7 was consistently elevated by JEV and reduced by SCFAs across both time points.

### SCFAs lead to a differential expression of microRNAs

**Fig 3:**
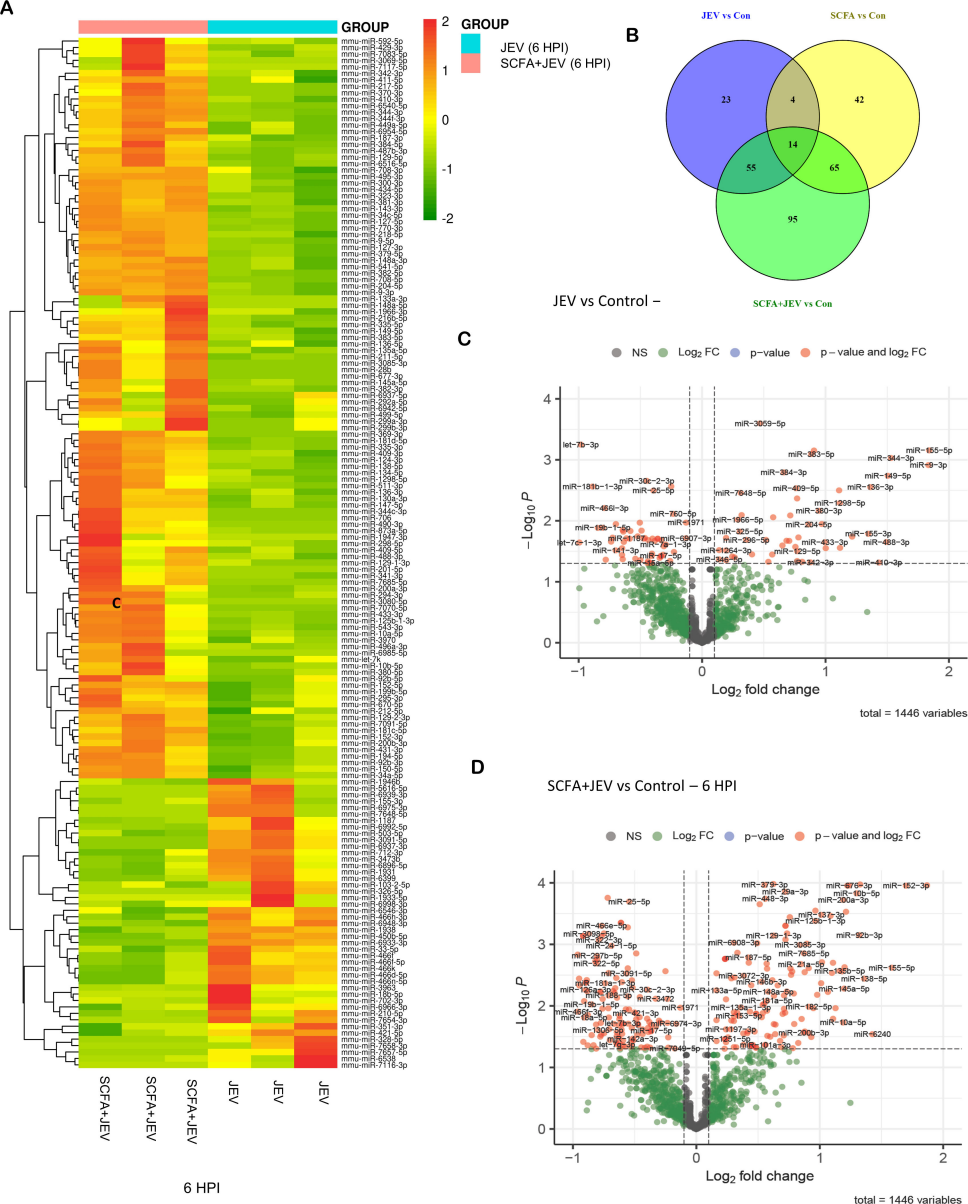
Differential expression analysis of microRNAs post SCFA pretreatment and/or JEV infection. Small RNA sequencing was performed post SCFA pretreatment and JEV infection at multipicity of infection (MOI) 3. (**A**) Represents heat map depicting differentially expressed microRNAs in JEV group and SCFA + JEV group 6 HPI. (**B**) Venn diagram showing the number of miRNAs differentially expressed across different conditions. (**C**) Volcano plot showing the differentially expressed miRNAs in JEV group versus control group. (**D**) Volcano plot showing the differentially expressed miRNAs in SCFA + JEV group versus control group.

Small RNA sequencing analysis revealed a total of 1,114 differentially expressed microRNAs (DE miRNAs) across all eight groups. miRNAs with ≥2 log fold changes and *P*-value <0.05 were identified as DE. Ninety-six miRNAs were DE (81 upregulated, 15 downregulated) between control and JEV 6 HPI (Fig. 3B). Between SCFA + JEV 6 HPI and control group, 220 miRNAs were DE (171 upregulated, 58 downregulated) (Fig. 3C). A heatmap was generated for the 160 DE miRNAs between JEV 6 HPI and SCFA + JEV 6 HPI (Fig. 3A). Similarly, between control and JEV 24 HPI, 60 miRNAs were dysregulated (44 upregulated and 16 downregulated). One hundred seventeen miRNAs were DE (90 upregulated and 27 downregulated) between control and SCFA + JEV 24 HPI. Heatmap was generated between 98 DE miRNAs between JEV 24 HPI and SCFA + JEV 24 HPI (Fig. S5). Differentially expressed miRNAs were further validated using quantitative real time polymerase chain reaction (qRT-PCR) (Fig. S6).

### Identification of miRNA networks and upregulation of miR-200a-3p in SCFA conditions

To comprehensively assess the co-expression patterns of miRNAs in our study, we employed weighted gene co-expression network analysis (WGCNA) across all eight experimental groups. This robust approach facilitated the identification of 18 distinct miRNA modules characterized by their differential expression under various conditions, as detailed in the Supplementary material. Notably, the “blue module” emerged as a significant point of interest, exhibiting a pronounced upregulation in response to SCFA conditions while displaying downregulation exclusively under JEV conditions (Fig. S7).

To delve deeper into the structure and relationships within these miRNA modules, we employed a dendrogram analysis. This hierarchical clustering method allowed us to visualize and understand the patterns of miRNA co-expression more intricately. The dendrogram, generated through hierarchical clustering, illustrates the co-expression relationships among miRNAs within each module, providing insights into their potential functional synchrony and divergence. The blue module, in particular, demonstrated a distinct clustering pattern, indicative of its unique regulatory role under varying conditions (Fig. S8).

In determining the optimal soft-thresholding power for network construction—a critical step in WGCNA—we conducted a softPower analysis. This analysis, essential for creating a scale-free topology, involved assessing the scale-free fit index for various soft-thresholding powers. The goal was to select a power at which the scale-free topology fit index reaches an acceptable threshold (typically 0.9), ensuring a balance between sensitivity and specificity in the network. The chosen softPower value informed the construction of a weighted adjacency matrix, integral to defining the strength of co-expression relationships between pairs of miRNAs (Fig. S9).

**Fig 4:**
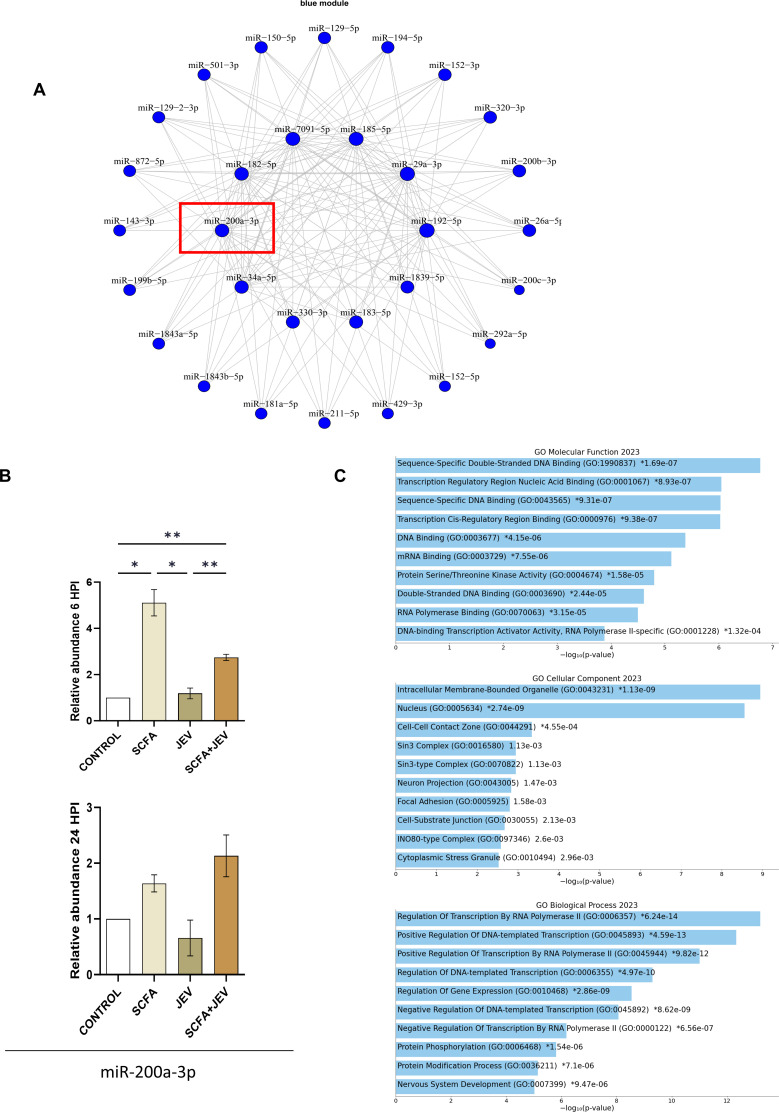
WGCNA revealed a network of miRNAs (blue module) significantly upregulated in SCFA conditions. (A) Represents the cluster of microRNAs color coded as the blue module that showed significant upregulation in SCFA conditions. (B) Bar graphs showing microRNA-200a-3p expression changes across all conditions at 6 HPI and 24 HPI. Data represented as mean fold change ± SEM with respect to control from a minimum of three independent experiments. *P*-values were determined (*, *P* < 0.05; **, *P* < 0.01; ***, *P* < 0.001) using one-way analysis of variance followed by Tukey’s *post hoc* correction. (C) Gene Ontology (GO) analysis graphs representing functional enrichment of the blue module at biological process, cellular component, and molecular function levels.

Mmu-miR-200a-3p, identified as a hub microRNA within the blue module (Fig. 4A), was further validated (Fig. 4B). This validation underscores the significance of mmu-miR-200a-3p in the context of SCFA and JEV conditions, potentially acting as a pivotal regulator. Additionally, functional enrichment and target prediction analyses of the blue module revealed a significant association with transcriptional regulation pathways (Fig. 4C). This finding suggests that the miRNAs in the blue module, particularly mmu-miR-200a-3p, may play crucial roles in modulating gene expression under the studied conditions.

### ZBTB20 is bioinformatically predicted as a target for miR-200a-3p

**Fig 5:**
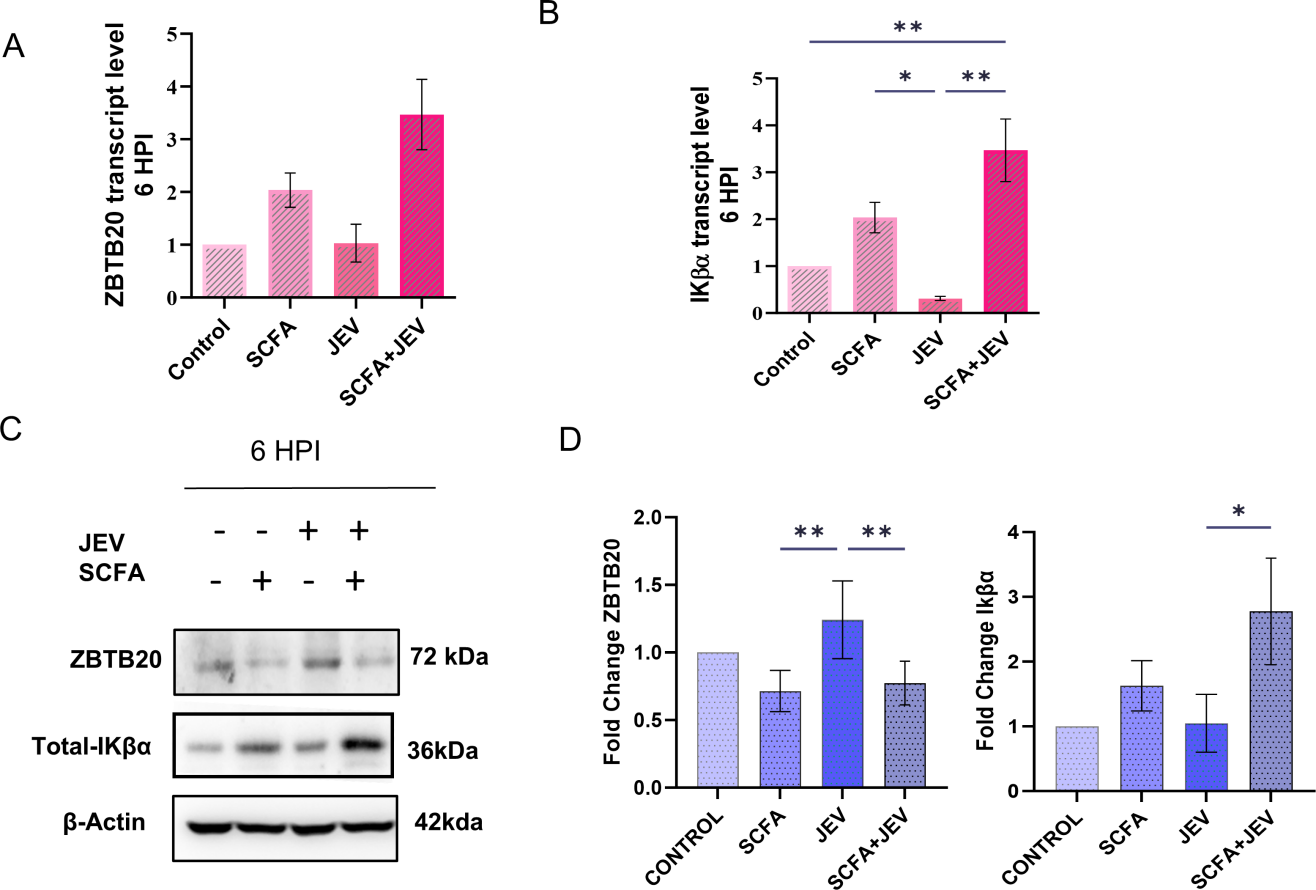
Post-transcriptional regulation of ZBTB20 leads to transcriptional regulation of Iκβα gene. (**A**) Bar graph representing mRNA expression levels of ZBTB20 gene across four conditions at 6 HPI. (**B**) Bar graph representing mRNA expression level of Iκβα across four conditions at 6 HPI. (**C**) Representable immunoblots of ZBTB20 protein from whole cell lysate after desired treatment paradigm using SCFA and/or JEV multipicity of infection [MOI] 3 at 6 HPI. Bar graphs showing the densitometric quantification of the immunoblots from the panel. Data represented as mean fold change ± SEM with respect to control from a minimum of three independent experiments. *P*-values were determined (*, *P* < 0.05; **, *P* < 0.01; ***, *P* < 0.001) using one-way analysis of variance, followed by Tukey’s *post hoc* correction.

**Fig 6:**
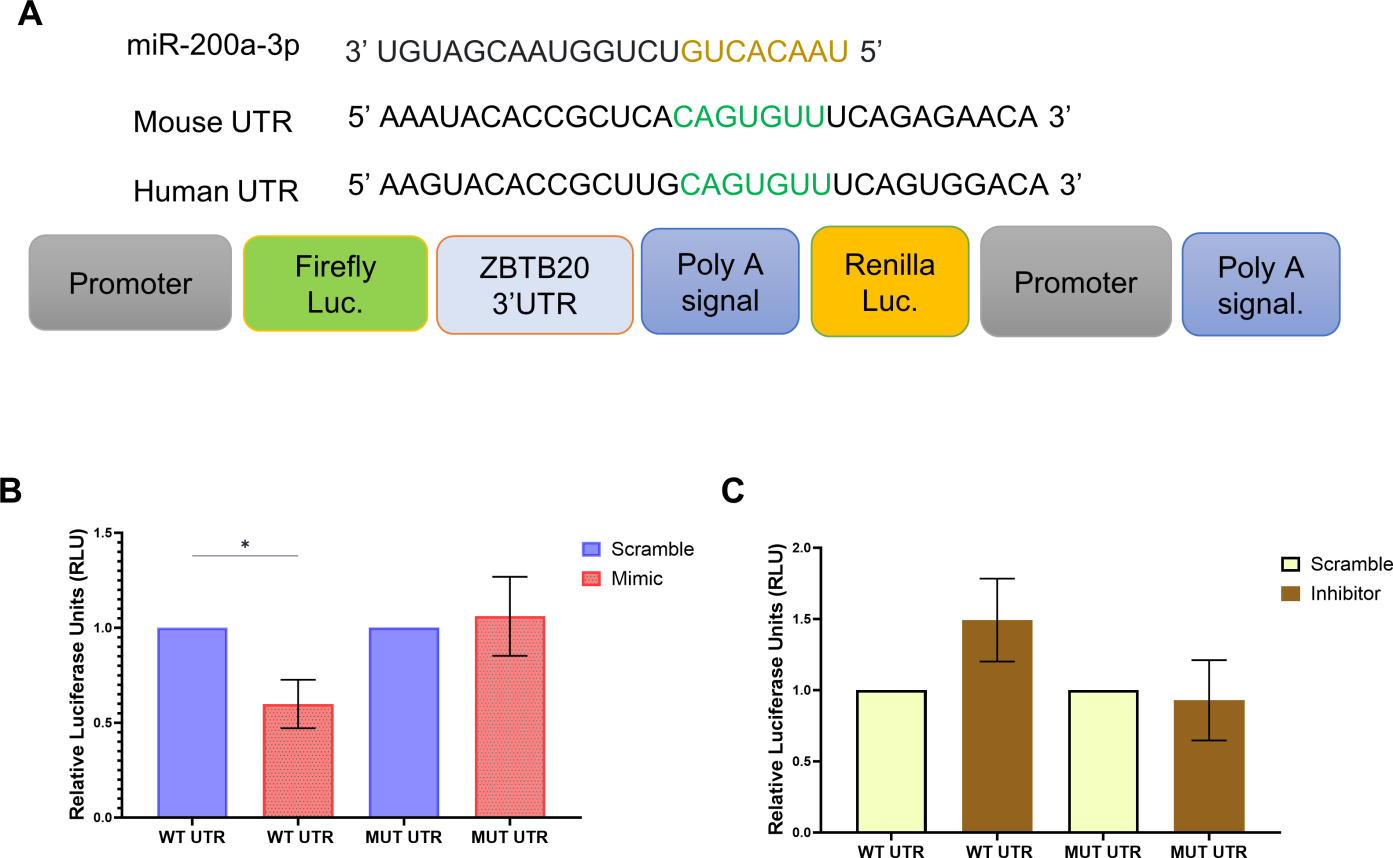
miR-200a-3p regulates ZBTB20 post-transcriptionally by targeting 3′ UTR region of ZBTB20 mRNA. (**A**) Illustration of the miR-200a-3p sequence, its seed region at 3′ UTR of ZBTB20, and the pmiRGlo construct carrying the 3′ UTR at its multiple cloning site. (**B**) Bar graph showing the dual luciferase activity of pmiRglo construct carrying the wild-type/mutated ZBTB20 UTR in the presence of scramble/miR-200a-3p mimic. (**C**) Bar graph showing the dual luciferase activity of pmiRglo construct carrying the wild-type/mutated ZBTB20 UTR in the presence of scramble/miR-200a-3p inhibitor. Data represented as mean fold change ± SEM with respect to control from a minimum of three independent experiments. *P*-values were determined (*, *P* < 0.05; **, *P* < 0.01; ***, *P* < 0.00) using Student’s *t*-test.

Employing various bioinformatics tools available online, we predicted ZBTB20 as a potential target for miR-200a-3p indicated by the seed sequence complementarity between miR-200-3p and ZBTB20 3′-UTR. Although mRNA analysis revealed no change in expression of ZBTB20 (Fig. 5A), significant reduction in protein expression was observed (Fig. 5C). Intuitively, a significant upregulation of IkBα at both mRNA and protein levels was observed (Fig. 5B and C), indicating a regulatory role of ZBTB20 TF toward IkBα at the transcriptional level. We observed no significant change at 24 HPI (Fig. S10). Hence, the following experiments were conducted at 6 HPI. To further confirm the interaction between ZBTB20 and mir-200a-3p, dual luciferase assay was performed. The assay revealed a reduction in luciferase activity when cells were co-transfected with ZBTB20 3′-UTR carrying plasmid and miR-200a-3p mimic (Fig. 6A). An inverse effect was observed with miR-200a-3p inhibitor. As evident in the graph, presence of miRNA inhibitor did elevate the luciferase activity of the UTR carrying plasmid, but was unable to reach a significant level when compared to the complementary scramble control. The effects of both miRNA mimic and inhibitor were alleviated when ZBTB20 3′-UTR was mutated using site-directed mutagenesis (Fig. 6B and C).

### ZBTB20 and IkBα orchestrate downstream regulation of NF-kB phosphorylation

**Fig 7:**
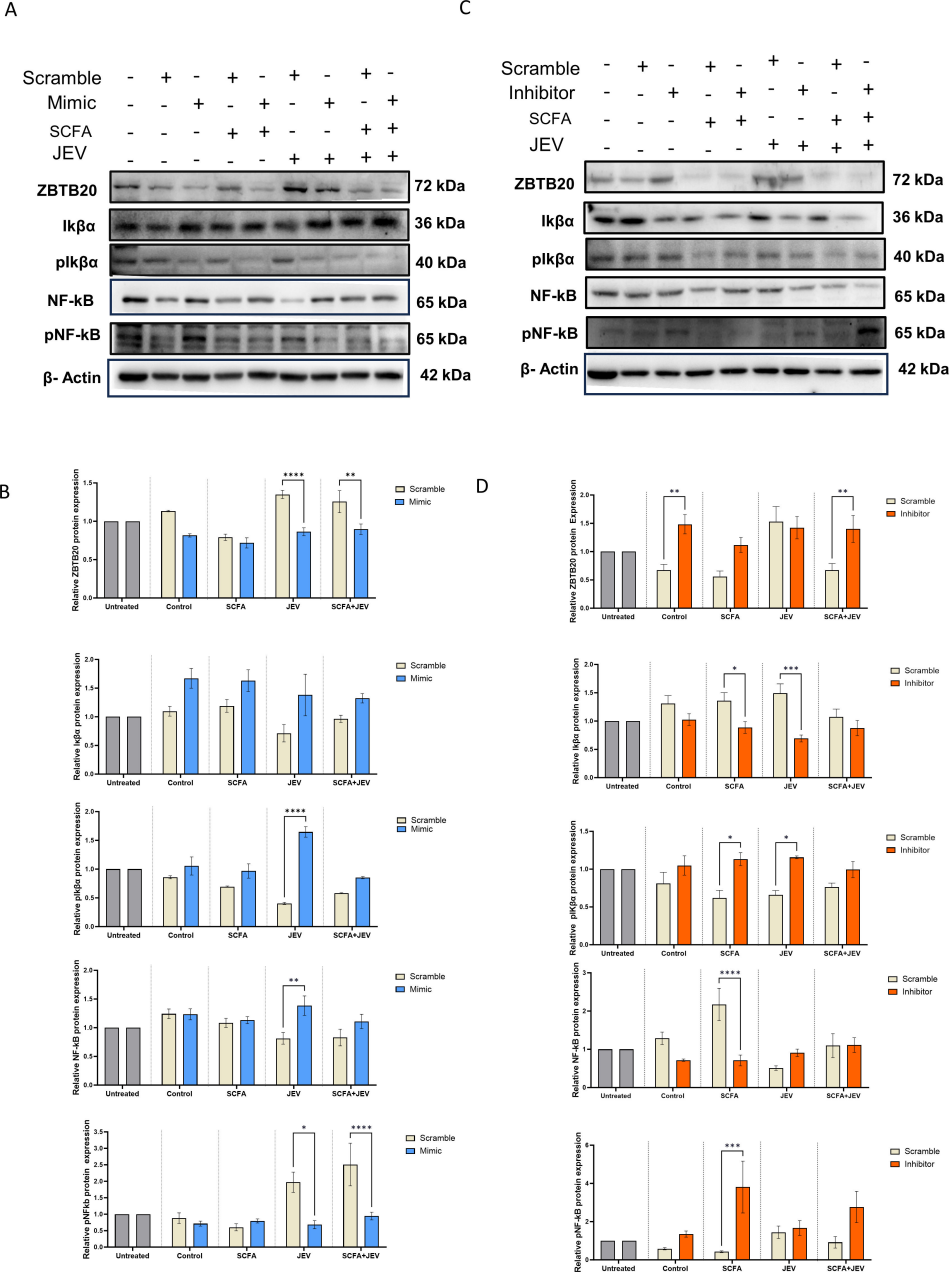
miR-200a-3p-regulated ZBTB20 alters Iκβα and NF-κB protein levels downstream. (**A**) Representable immunoblots showing protein expression of ZBTB20, total/p-Iκβα, and total/pNF-κB in the presence of scramble/miR-200a-3p mimic in untreated, SCFA, JEV, and SCFA + JEV conditions. Each mimic condition has been compared to its scramble counterpart. (**B**) Bar graphs represent densitometric quantification of protein levels from panel A. (**C**) Representable immunoblots showing protein expression of ZBTB20, total/p-Iκβα, and total/pNF-κB in the presence of scramble/miR-200a-3p inhibitor in untreated, SCFA, JEV, and SCFA + JEV conditions. Each inhibitor condition has been compared to its scramble counterpart. (**D**) Bar graphs represent densitometric quantification of protein levels from panel C. Data represented in grouped form as mean fold change ± SEM with respect to scramble control in each condition. A minimum of three independent experiments were used. Scramble controls have been normalized using untreated control group. *P*-values were determined (*, *P* < 0.05;**, *P* < 0.01;***, *P* < 0.001) using two-way analysis of variance and Tukey’s *post hoc* correction.

We conducted mimic/inhibitor assay to observe a possible relationship between the miR-200a-3p target ZBTB20 and key proteins of the NF-κB signaling pathway, namely total/phospho(p)IkBα and total/pNF-κB. In conditions where miR-200a-3p was overexpressed using mimic, or using SCFA, a downregulation of ZBTB20 was observed. ZBTB20 is observed to be higher in the scramble mimic (ScrM) subgroup when compared to untreated control (UC). This level is brought down by mimic as observed in the mimic subgroup, even though this decline is not statistically significant. SCFA treatment, as expected, kept the ZBTB20 levels lower than UC as shown in the SCFA group. Both scramble and mimic subgroup showed a decreased level of protein due to the presence of SCFA (positive control for the mimic). The JEV + scramble mimic subgroup had upregulated ZBTB20 levels which were brought down significantly in the JEV + mimic subgroup. A similar significant decline was observed in SCFA + JEV + mimic subgroup when compared to its scramble control. This was accompanied by upregulated or unchanged total IKβα and NF-κB across four conditions. Only NF-κB in the JEV + mimic subgroup was upregulated significantly. A consequent decline in pNF-κB was also observed significantly in the JEV and SCFA + JEV groups compared to their respective scramble controls. Interestingly, the scramble/mimic and SCFA group also had low levels of pNF-κB (Fig. 7A and B). Despite total IKβα levels going up, we did not observe a significant shift of the pIKβα upon densitometric analysis. The levels in the SCFA and SCFA + JEV group stayed statistically unchanged. It was in the JEV group that we observed an unexpected decline in the pIKβα in the scramble subgroup and a rise in the JEV mimic subgroup, which did not correlate to the pNF-κB levels in the same conditions. In the second leg of this experiment, inhibition of miR-200a-3p using an inhibitor or JEV led to an upregulated ZBTB20 across all groups with significant change in the inhibitor and SCFA + JEV + inhibitor subgroups; this upregulation was similar to the upregulation caused by JEV (positive control for the inhibitor). Furthermore, downregulated total IKβα and NF-κB were observed in all groups with a significant decline in the SCFA and JEV conditions. Expectedly, pIKβα and pNF-κB were upregulated in all inhibitor subgroups with significance in the SCFA and JEV subgroups. The inverse rise and fall of total IKβα, pIKβα and NF-κB are evident by the densitometric analysis of SCFA and JEV subgroups (Fig. 7C and D).

## DISCUSSION

Invasion of CNS by neurotropic flaviviruses elicits an innate immune response as the frontline host defense. Among these viruses, JEV induced neuroinflammation and neuronal death has been extensively studied (5, 29, 30). In the brain, JEV infection leads to microglia activation and subsequent release of cytokines and chemokines as effector molecules. Activation of NF-κB and secretion of TNFα, IL-6, and MCP-1 (CCL2) are hallmark features of a proinflammatory environment. In this study, N9 microglia as the *in vitro* cell model robustly showcased JEV-mediated inflammation (Fig. 1). Complex microbiota in the gut translate their functionality via metabolites such as SCFAs. The significant reduction in MCP1 and TNFα when N9 cells were pretreated with SCFAs (Fig. 1B) solidifies the latter’s known ability to regulate immune response via free fatty acid receptors (FFARs) or HDACs (11, 31) . In 2023, Caetano-Silva et al. demonstrated the downregulation of TNFα, IL1β, and IL10 in LPS-stimulated primary microglia when pretreated with acetate and butyrate. They attributed this observation to HDAC inhibition, independent of FFAR2 as microglia do not express these receptors (32). When we checked for a possible reduction in viral load in SCFA-pretreated conditions, the threefold reduction in the viral NS3 at 24 HPI (Fig. 1C and D) indicated a potential antiviral role of SCFAs. However, the viral NS3 protein is undetectable at early time points such as 6 HPI. Hence, viral RNA (GP78) detection by qRT-PCR was employed. We observed a statistically insignificant but consistent decrease in viral RNA in SCFA-pretreated conditions (Fig. S4), a finding that warrants further investigation. As we use a combination of three SCFAs in this study to mimic physiological proportions to an extent, the effect observed cannot be delineated to any one SCFA.

Interestingly, we observed a significant reduction in whole cell HDAC protein levels as well as HDAC activity upon SCFA treatment (Fig. 2). There are multiple reports of reduction in activated NF-κB due to HDAC inhibition (33–36). In 2015, Lin et al., in their study, demonstrated butyrate and propionate inhibited HDAC activity by changing chromatin structure. They suggested specific regulation of HDACs by each individual SCFA to initiate a tailored Toll-like receptor (TLR) response in intestinal epithelial cells (37).

Building upon initial observations, we posited that SCFAs could modulate microglial function by intricately adjusting their miRNA profiles, thereby influencing key inflammatory pathways. Previous studies have established the pivotal role of miRNAs in a host of cellular mechanisms, especially in the context of flavivirus infections (38). A pertinent question for future research is whether the shifts we observed in miRNA expression profiles and their downstream effects are attributable to HDAC inhibition.

In our study, DE of miRNAs was evident when cells were pretreated with SCFAs before JEV infection lasting 6 HPI and 24 HPI (Fig. 3; Fig. S5). Among these DE miRNAs in the JEV 6 HPI group, miR-155 was notably upregulated post-infection, corroborating with previous findings in mouse primary cells where it repressed SHIP1, leading to increased proinflammatory cytokine production (25). In human microglia, upregulated miR-155 has been observed to constrain JEV replication, suggesting a protective role for the host cell (39). However, contrasting reports indicate miR-155-5p’s modulation of its target PEL-1 facilitates JEV in evading the host’s immune response (40). Notably, miR-155 was downregulated by 1.41-fold in the SCFA + JEV 6 HPI group.In the SCFA + JEV 6 HPI group, we noted significant downregulation of miR-19b-5p (Fig. 3C). While miR-19b-3p’s upregulation has been associated with JEV-induced inflammation (41), the role of miR-19b-5p remains less explored. Furthermore, miR-181b-1-3p, significantly downregulated in our data, is known to regulate NF-κB activation via targeting importin-α3, which assists in NF-κB nuclear translocation in vascular endothelial cells (42). miR-141, implicated in targeting SIRT1 and influencing autophagy in hepatitis B virus *in vitro* models, along with miR-129-5p, which regulates the microglial surface receptor CD200R1 to control neuroinflammation (43), were also observed to be DE in our study.

miR-9-5p, typically downregulated in the presence of JEV in neural stem cells, was significantly upregulated in SCFA + JEV 6 HPI group. The induction of miR-369-3p, known to mitigate chronic inflammatory responses (44), and miR-708, which negatively regulates TNFα/IL1β to suppress NF-κB activation (45), were also noted. Furthermore, miR-152 overexpression as observed in our data has been previously recorded in BV2 microglia showing an anti-inflammatory response (46). Repressed miR-34c-5p expression in JEV-promoted NOTCH1 signaling, and cytokine production (18) was reversed in SCFA + JEV groups. In addition, we endeavored to elucidate the microRNA networks operational under our experimental conditions. Post WGCNA, we identified a cluster of miRNAs (the blue module) significantly upregulated specifically in SCFA conditions. This finding spurred further investigation into this module. Target and functional enrichment of this module reveals the member miRNAs being strongly associated with transcriptional regulation. This indicates the role of transcription activators and repressors that need further investigation. MicroRNA-200a-3p, a hub miRNA within this module and differentially expressed in SCFA + JEV conditions, was selected for detailed analysis (Fig. 4). Emerging evidence suggests a diverse role for miR-200a-3p in multiple disease states. Wang et al. reported that miR-200a-3p, suppressed in Alzheimer’s disease models, when supplemented, led to reduced cell apoptosis, diminished amyloid beta-peptides deposits, and decreased tau phosphorylation (47). This aligns with literature suggesting a protective effect conferred by the miRNA (48–50), although its deleterious impact in various cellular contexts is also documented (51–53). The differential effects of miR-200a-3p on host cells vary with respect to human papillomavirus (HPV) infection and cancer cell types (54). HDAC 3 inhibition has been shown to upregulate miR-200a-3p, regulating KEAP1 protein and preserving hepatic FGF21 synthesis in diabetic mice (55). An inverse relationship between PELP1 and miR-200a regulated by HDAC 2 activity (56) and a feedback loop between HDAC 4 and miR-200a in hepatocellular carcinoma (57) highlight the potential impact of HDAC changes on miRNA profiles, including miR-200a-3p. Assessing HDAC protein binding to the miR-200a-3p promoter could provide clarity on this hypothesis in future.

This study is the first to explore miR-200a-3p mechanisms in the context of neurotropic virus infection. ZBTB20, predicted as a target of miR-200a-3p, is a known repressive TF (58). Downregulation of ZBTB20 in a mouse model aimed at preventing allograft rejection led to increased FOXP3 gene expression and alleviated NF-κB-mediated inflammation, prolonging heart transplant survival (59). Liu et al. demonstrated ZBTB20’s binding to the IKβα gene promoter, regulating its transcription and downstream NF-κB activation. Knockout of ZBTB20 reduced TLR-mediated immune responses triggered by bacterial toxins. In our study, we add another layer of regulation of this TF by miR-200a-3p, demonstrating the direct interaction between miR-200a-3p and ZBTB20 via a dual luciferase activity assay. Using a miRNA mimic, we show how SCFA-mediated miR-200-3p and ZBTB20 interaction leads to increased total IKβα protein levels and reduced NF- κB activation, an effect reversed in the presence of a miR-200a-3p inhibitor, thus reinforcing this relationship.

Recently, we undertook a study to investigate the impact of SCFA intraperitoneal supplementation prior to JEV infection in a mouse model. We observed a significant reduction in microglial activation status, inflammatory proteins, and neuronal death in mice pretreated with SCFAs (60). This led us to focus our attention on the molecular mechanisms that potentially underlie the effect of SCFAs on microglial cells. Hence, we demonstrate SCFAs regulating the HDAC expression and activity that in turn appear to change the microRNA expression pattern of JEV-infected host microglial cells *in vitro*. However, this study takes a reductionist approach to understanding a physiologically complex interplay between the gut and brain. The role of SCFA is a double-edged sword that can be used for and against the host cell depending on the context. The prior supplementation of SCFAs such as acetate has been well-recorded as a protective feature in microglial function in developing animal model (61). In order to establish the double edged-sword effect, another recent publication from our group reported the proviral role of a single SCFA, butyrate when supplemented post-JEV inoculation in animal and neuronal cell model (62). In the current study, we made a limited observation of the viral RNA load when the cells have been treated with SCFA post-JEV inoculation as opposed to pre-SCFA treatment. Here, we did not observe any statistically significant trend in JEV mRNA level in this paradigm (Fig. S2). Whether SCFAs can be utilized as a treatment instead of a preventative tool remains a gray area and needs elaborate investigation.

It is imperative to understand whether the JEV host system experiences an alteration in the gut microbiota and SCFA levels in the presence of viral infection. Alternatively, do alterations in microbiota taxa and SCFA levels make an organism more susceptible to JEV neurotropism? These are some questions that we are interested in addressing in future studies. Further validations of these mechanisms in animal model, where the gut microbiome can also be studied in parallel to JEV-infected CNS, can strengthen our evidence even further. Translation of our *in vitro* findings would be most beneficial if, in the future, we can prevent an organism from experiencing deleterious viral disease by prior metabolite supplementation or through dietary interventions.

## MATERIALS AND METHODS

### Animals

BALB/c mice were procured from Jackson Laboratory, USA, and were inbred and housed in a pathogen-free environment in controlled temperature and humidity. They were maintained in a 12-h day/night cycle; food and water were provided as required.

### Cell culture

Mouse microglial cell line N9 was a kind gift from Prof. Maria Pedroso de Lima, Center for Neuroscience and Cell Biology, University of Coimbra, Portugal. The cells were grown in Roswell Park Memorial Institute medium (RPMI-1640) supplemented with 10% fetal bovine serum (Sigma-Aldrich, USA), penicillin (100 U/mL), and streptomycin (100 µg/mL) and were incubated at 37°C and 5% CO_2_. All the reagents used for cell culture were purchased from Gibco, unless specified otherwise.

### Virus propagation and titration

All the experiments were performed using strain GP78, an Indian strain of JEV isolated during an encephalitis breakout in Gorakhpur in 1978 from a female post mortem brain sample (63). The virus was propagated and quantified according to a previous well-established protocol in our laboratory (64).

### Viral load and entry assay

In order to observe viral load during our experiments, we used either NS3/Plaque/GP78 . Viral titers cannot be assessed using plaque assay/western blot at early time points. Hence, viral mRNA GP78 was analyzed using qRT-PCR (Table 1) (detailed in Fig. S4). Furthermore, to observe possible changes in the infectivity of the virus in the presence of SCFAs, mimic, inhibitor, and their respective controls, a viral entry assay was performed (detailed in Fig. S3).

**TABLE 1:** List of primers used^*a*^

Target		Sequence (5′ to 3′)
JEV-GP78	Sense	TTGACAATCATGGCAAAC
	Antisense	CCCAACTTGCGCTGAATAA
ZBTB20	Sense	CCTCATCCACTCGACACATTCAC
	Antisense	GAAGGTTGATGCTGTGAATGCGC
IKβα	Sense	GCCAGGAATTGCTGAGGCACTT
	Antisense	GTCTGCGTCAAGACTGCTACAC
mmu-miR-122–5p		TGGAGTGTGACAATGGTGTTTG
mmu-miR-1946b		GCCGGGCAGTGGTGGCACATGCTTTT
mmu-miR-1a-3p		TGGAATGTAAAGAAGTATGTAT
mmu-miR-31–5p		AGGCAAGATGCTGGCATAGCTG
mmu-miR-499–5p		TTAAGACTTGCAGTGATGTTT
WT ZBTB20	Sense	TCAGCA**GAGCT**CGCTACTGGCCTCTAAATACAACC
	Antisense	ACTGCT**TCTAGA**CCCACCCTCCATTTCTTTTTCT
SDM ZBTB20	Sense	ACACCGCTCA**CA****GT****TTT**CAGAGAACAGTT
	Antisense	AACTGTTCTCTG**AAA****AC****TG**TGAGCGGTGT
Plasmid-specific primer (Luc)	Sense	AAGGGCGGCAAGATCGCCGTGTAA

^*a*^ WT, wild type; SDM, site-directed mutagenesis.

### Treatment paradigm

N9 microglial cells were seeded and serum starved for 2 h and, thereafter, treated with either control media or SCFA cocktail (sodium acetate:sodium butyrate:sodium propionate—12 mM:800 µM:400 µM) in 2% fetal bovine serum (FBS) substituted RPMI for a span of 12 h. Furthermore, the SCFA containing media were removed and the cells were serum starved for 2 h, followed by incubation with virus (3 MOI)/serum-free media for 2 h and the virus containing media replaced with maintenance media (2% FBS + RPMI) until collection. The cells were collected at 6 HPI and 24 HPI for further experiments. This study has four conditions in the basic paradigm—untreated control (C), only SCFA treated (SCFA), only JEV infected (JEV), and SCFA treated + JEV infected (SCFA + JEV) (Fig. 1A). The SCFA dosage and treatment time were fixed after subjecting N9 to an MTS assay, a colorimetric based assay for determining cell viability (Fig. S1A). The dosage used in this study was responsible for the most significant suppression of cytokines in the presence of JEV based on an initial pilot study (data not shown). Additional cell viability assay was also conducted along the entire treatment paradigm (Fig. S1B) and mimic/inhibitor transfection protocols (Fig. S1C).

### Cytokine bead array

CBA (Cytometric Bead Array mouse inflammation kit, cat no. 552364, BD Biosciences) kit was used to quantify the mature secreted cytokine from the cell supernatant according to the manufacturer’s protocol . The amount of cytokine was measured using BD FACS verse and analyzed using FCAP array 3.0.

### Small RNA sequencing

Cell samples in TRIZOL were outsourced to Clevergene Biocorp Private Limited, Bangalore, for performing small RNA sequencing. Total RNA was extracted using phenol-chloroform method. The RNA pellet was dissolved in RNase free water and rRNA depletion was performed using 1 µg of total RNA (NEBNext rRNA Depletion Kit, New England Biolabs) according to manufacturer’s protocol. NEBNext Ultra II Directional RNA Library Prep Kit was used to prepare RNA-seq libraries, and the sequencing data were generated using Illumina HiSeq. Targeted read generation was 10–15 million. Quality check of the data was performed using FastQC v.0.11.9 and MultiQC v.1.9 software. Trimgalore v.0.6.6 and Cutadapt v.3.4 programs were used to remove adaptor sequences, low-quality bases, and reads shorter than 14 bp. Post all desired quality check, the sequencing data were mapped onto indexed mouse reference genome (GRCm38) using mapper.pl script of miRDeep2 v.2.0.1.2.

### Differential expression analysis

DESeq 2 package was used to perform differential expression analysis. Post normalizing the read counts, the test samples were compared to the control samples. miRNAs with absolute log2 fold change ≥ 1 and *P*-value ≤ 0.05 were considered significant. The expression profile of the differentially expressed miRNAs across the samples is presented in volcano plot and heat map.

### WGCNA analysis

We constructed miRNA co-expression networks utilizing the R package WGCNA, following the methodology previously described (65). In this process, a thresholding power of 12 was selected, which represented the minimal threshold that achieved a scale-free *R*^2^ fit of 0.8. This choice ensured that the network adhered to the scale-free topology criterion, a key aspect in the robust construction of co-expression networks.

To construct the network, we computed the component-wise minimum values of topological overlap, a measure that reflects the interconnectedness of miRNAs within the network. Subsequently, miRNAs were subjected to hierarchical clustering based on this topological overlap, facilitating the identification of closely related groups of miRNAs.

The initial module assignments within the network were determined using a dynamic tree-cutting algorithm. For this, we employed the “cutreeHybrid” function, adhering mostly to default parameters but with specific modifications: deepSplit set to 4, cutHeight at 0.999, a minimum module size of 40, dthresh at 0.2, and pamStage disabled. These parameters were carefully chosen to optimize the detection and delineation of miRNA modules within the network.

### Target prediction, plasmid construction, and dual luciferase assay

Freely available software, TargetScan Mouse 8.0, was used to predict candidate target genes of miRNA-200a-3p. ZBTB20 was predicted with four conserved binding sites for the microRNA. Hence, we went on to clone a segment of ZBTB20 3′ untranslated region of messenger RNA (UTR) into pmirGlo reporter plasmid (Promega). A 785 bp segment of the 3′ UTR of mouse ZBTB20 (carrying a miR-200a-3p binding site) was amplified by PCR from mouse cell line N9 cDNA (Table 1). The DNA fragment was inserted between XbaI and SacI sites in the multiple cloning site downstream of the firefly luciferase gene. Site-directed mutagenesis performed with the site-directed mutagenesis (SDM) primers (forward and reverse; Table 1) generated mutant clones. A PCR-based method involving Phusion High Fidelity DNA Polymerase and DpnI (New England Biolabs) was performed for mutagenesis. The presence of ZBTB20 insert in wild-type and mutant plasmids was confirmed using a combination of vector-specific luc-ZBTB20 forward primer and insert-specific wild-type ZBTB20 (WT-ZBTB20) reverse primer. Subsequently, the WT and SDM constructs were confirmed by Sanger sequencing at Regional Centre for Biotechnology, Faridabad, India.

### Transfection of cells using miRNA mimic/inhibitor

To overexpress or inhibit the microRNA expression in N9 cells, a small RNA molecule mimicking/inhibiting mature miR-200a-3p or a respective scramble miRNA was transfected using optiMEM media. The cells were further subjected to SCFA pretreatment and JEV infection as mentioned in “Identification of miRNA networks and upregulation of miR-200a-3p in SCFA conditions.” Samples were collected at 6 HPI and labeled as follows: control (C), scramble mimic/inhibitor (ScM/ScI), mimic/inhibitor (M/I), S + ScM/ScI, S + M/I, J + ScM/ScI, J + M/I, S + J + ScM/ScI, S + J + M/I.

### RNA isolation and PCR analysis

Total RNA from cells was isolated using TRIZOL method (TRI reagent, Sigma Aldrich). Five hundred nanograms of RNA was converted to miRNA-specific cDNA using miRNA 1st-strand cDNA synthesis kit (Agilent Technologies) as per manufacturer’s protocol. mRNA-specific cDNA was prepared using Verso cDNA kit (Thermo scientific). For validation experiments, miRNA-specific primers were carefully designed using primerquest tool of IDT DNA according to the universal primer designing protocol. Target gene expression was detected by qRT-PCR using mRNA-specific primers. All primer sequences are provided in Table 1. iTaq Universal SYBR Green Supermix was used to perform real-time PCRs according to the manufacturer’s protocol. Relative abundance of miRNAs was calculated using dCt method after normalizing the Ct values of the miRNA of interest with U6. All the PCRs were performed in QuantStudio-5 Real time PCR system (Thermo Fisher Scientific). Expression analysis of miRNA-200a-3p was performed using miRCURY LNA miRNA PCR assay (Qiagen, Germany) as per manufacturer’s protocol.

### Immunoblotting

Protein isolation was done from N9 microglial cells post desired treatment paradigm based on our previous protocol (66). The amount of protein was quantified using bicinchoninic acid reagent.

Equal amounts of protein were separated using SDS-PAGE and transferred to nitrocellulose membrane. The blot was then blocked using either 10% milk or 10% bovine serum albumin (BSA) for 3 h, post which the membrane was incubated in primary antibody against the protein of interest overnight at 4°C: anti-NS3 (cat no. GTX125868, 1:10,000 Genetex), anti-Iba1 (cat no. 019-19741, 1:1,000, Wako), anti-ZBTB20, anti-phospho-IκBα, anti-total IκBα, anti-phosho-NF-κB, anti-total NF-κB, and anti-β-actin. Subsequently, the membrane was washed using 1× Tris-buffered saline with 0.1% Triton X-100(TBXT) and incubated in appropriate secondary antibody at room temperature (RT) for 2 h. Thereafter, the membrane was washed and visualized using Immobilon western chemiluminescent HRP substrate (cat no. WBKLS0100, Millipore, Sigma-Aldrich) in Uvitec Cambridge (Cleaver Scientific, UK). Images of immunoblots were taken with Chemigenius Bioimaging System (Uvitech Cambridge). Each blot was normalized with β-actin (after stripping and re-probing with the β-actin antibody). Software ImageJ was used for analysis of the blots.

### HDAC activity assay

N9 microglial cells were seeded in a 96-well plate; upon reaching desired confluency, the cells were serum starved for 2 h, post which the cells were subjected to either of the three conditions: SCFA cocktail treatment, trichostatin A, and negative control. HDAC Glo I/II assay (Promega) was employed according to the manufacturer’s protocol to analyze the HDAC activity. Readings were taken in Glomax luminometer (Promega) as per manufacturer’s protocol 30 min and 12 h post the treatment.

### Statistical analysis

Statistical analysis was performed using GraphPad Prism 9.0 software. Data were analyzed using appropriate statistical tool. One-way analysis of variance (ANOVA) was performed, followed by Tukey’s *post hoc* test to evaluate statistical significance between four groups. Two-way ANOVA was applied to calculate significance in grouped data, where each condition was grouped in accordance to two variables, followed by Tukey’s *post hoc* test. Student’s *t*-test (two-tailed) was performed for evaluating statistical significance between two groups.

## Data Availability

Raw and processed data files from small RNA sequencing have been deposited in GEO data sets (NCBI) under the accession number GSE267173.

## SUPPLEMENTAL MATERIAL

Supplemental figures mbio.01321-24-s0001.docx Fig. S1 to S10.

ASM does not own the copyrights to Supplemental Material that may be linked to, or accessed through, an article. The authors have granted ASM a non-exclusive, world-wide license to publish the Supplemental Material files. Please contact the corresponding author directly for reuse.
