# Supplementary material for: Short-chain fatty acids abrogate Japanese encephalitis virus-induced inflammation in microglial cells via miR-200a-3p/ZBTB20/IKβα axis
Source: mBio. 2024 Jun 13;15(7):e01321-24. doi: 10.1128/mbio.01321-24 (PMC11253640; doi:10.1128/mbio.01321-24)
Supplement: Supplemental figures — Fig. S1 to S10. [file mbio.01321-24-s0001.docx]

**SUPPLEMENTARY INFORMATION**


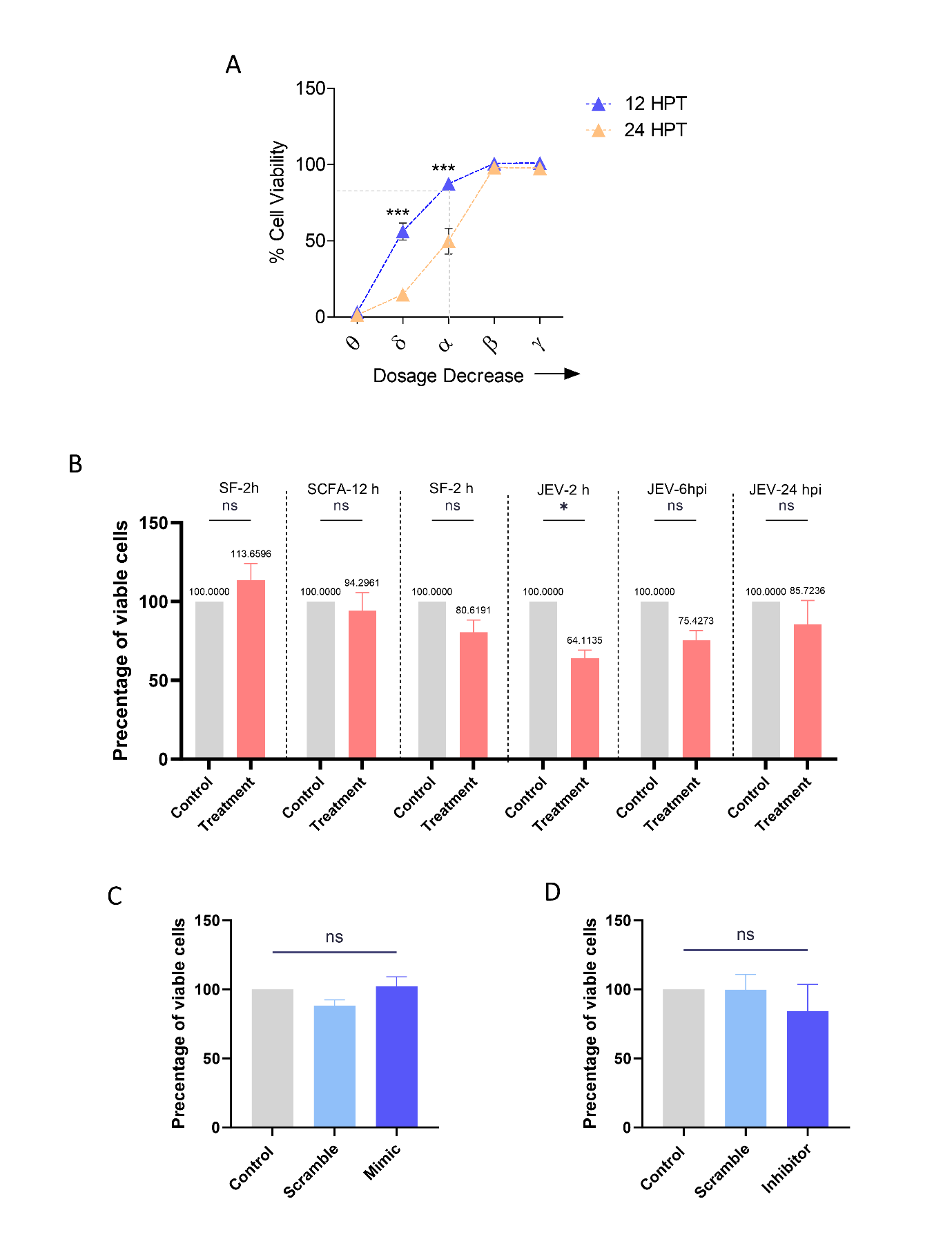


**Figure S1 – Cell viability assay at different concentrations of SCFA cocktail**

**Method-** N9 cells were seeded in 96 well plate until appropriate confluency was reached. Cells were subjected to (A) SCFA Dosage treatment [Five dosages of SCFAs were assessed, namely, γ-(acetate:propionate:butyrate-300uM:20uM:10uM), β-(acetate:propionate:butyrate-3mM:200uM:100uM), α-(acetate:propionate:butyrate-12mM:800uM:400uM), θ-(acetate:propionate:butyrate-48uM:3.2uM:1.6uM), δ-(acetate:propionate:butyrate-192mM:12.8mM:6.4mM)],(B) JEV and SCFA treatment paradigm (to begin with, six groups of cells were given the first step of treatment i.e. serum free (SF) media incubation, following which one group was subjected to MTS assay while the remaining five groups proceeded to the next stage of treatment with an MTS assay recording at each subsequent time point) and (C) Mimic/Inhibitor transfection. To assess cell viability after each treatment, CellTiter 96 Aqueous One Solution Cell Proliferation assay (MTS) (Promega, USA) was performed according to the manufacturer’s protocol. Data plotted as mean±SEM and two-way ANOVA was performed. Tukey’s post hoc test was employed for analyzing significance, p<0.05 was considered statistically significant.

**Result –** (A)The plot shows the percentage of cells surviving post different SCFA cocktail doses at 12 hours post treatment (HPT) and 24 HPT. Dosage θ led to the least cell viability percentage at both time points. Dosage δ led to 50% viability at12 HPT and <10 % at 24HPT. Dosage α β, γ allowed cell viability of >85% at 12HPT. **(B)** The plot shows the percentage of cells surviving at each time point during SCFA and JEV treatment paradigm. A brief dip in viability (64.1%) was observed at the 4^th^ time point, with the cells recovering in the next stage. No other significant change was observed in cell viability throughout the paradigm. (C-D) The plot shows the percentage of cells surviving after mimic/inhibitor/scramble transfection protocol with no significant changes across any condition


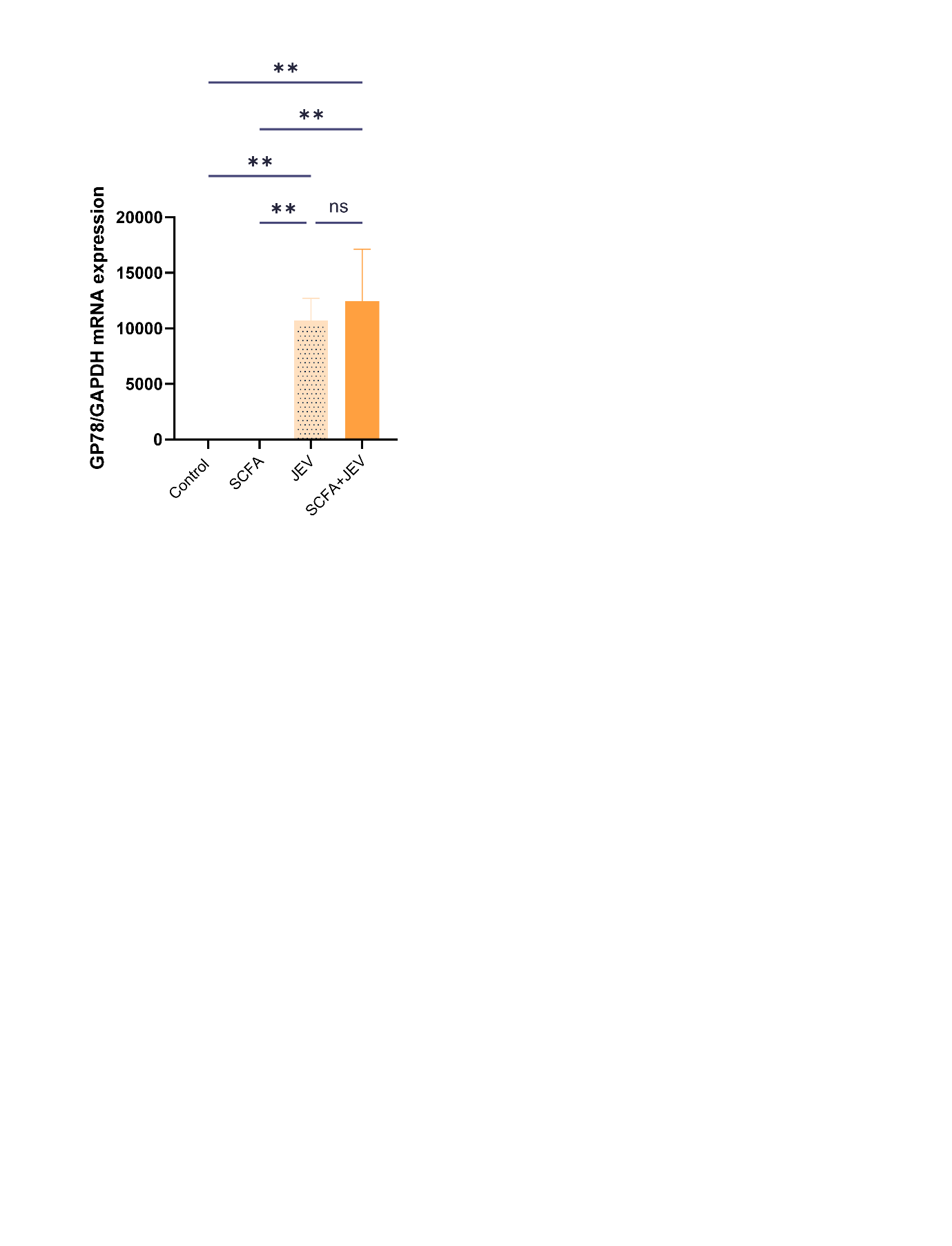


**Figure S2 – Viral RNA levels in SCFA treatment post JEV infection.**

**Method -** To assess if SCFA treatment post JEV infection had any effect on the viral mRNA level, N9 microglial cells were serum starved for 2 hrs, infected with JEV at 3 MOI followed by SCFA treatment. Cells were collected 6 hpi and PCR analysis against viral RNA was performed. Data plotted as mean fold change ± SEM. One-Way ANOVA followed by Tukey’s post hoc test was performed for analyzing significance, p<0.05 was considered statistically significant.

**Result** - The bar graph shows mean fold change of 10,705 in JEV condition versus 12,444 in SCFA+JEV condition. No significant change was observed in viral RNA levels when cells were treated with SCFA after JEV inoculation.


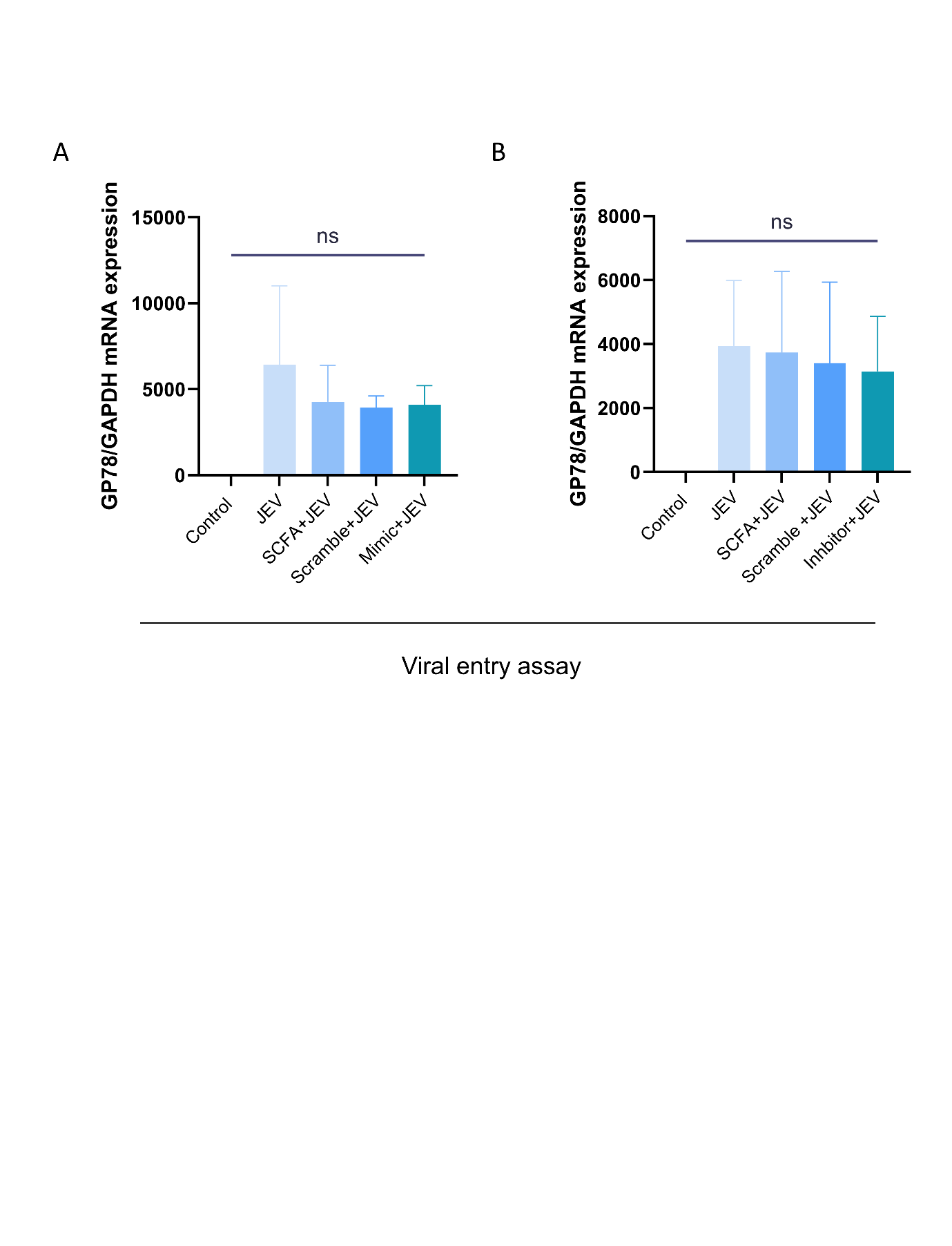


**Figure S3 – Viral entry assay with scramble and mimic/inhibitor transfection**

**Methods** – To understand if any treatment had an effect on the entry of JEV, N9 microglial cells were transfected with scramble/mimic/inhibitor followed by viral infection at 30 MOI and the cells were collected 1 h post infection. qRT PCR was performed to quantify the viral RNA copies just post entry. Data are represented as mean fold change ± SEM. One Way ANOVA followed by Tukey’s post hoc test was performed and p<0.05 was considered statistically significant.

**Results** (A) Fold change of viral RNA expression post mimic/scramble transfection – No significant change in the viral entry was observed as seen from the viral mRNA levels post mimic transfection

(B) Fold change of viral RNA expression post inhibitor/scramble transfection - No significant change in the viral entry was observed as seen from the viral mRNA levels post inhibitor transfection


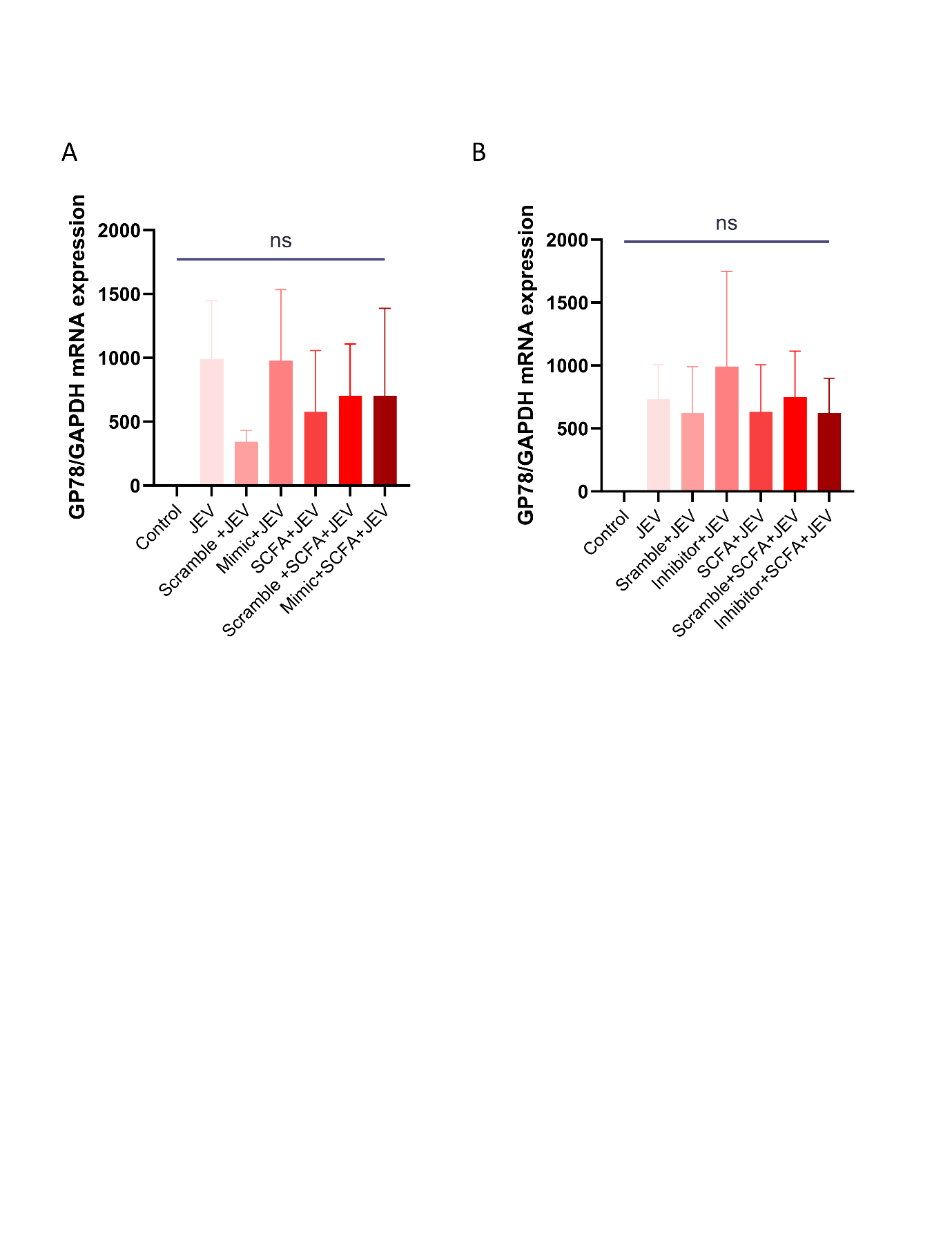


**Figure S4 – Viral mRNA expression post miRNA200a-3p inhibitor and mimic paradigm**

**Methods –** N9 cells were transfected with either mimic/inhibitor/scramble followed by SCFA treatment and JEV infection at 3 MOI. qRT-PCR analysis against viral RNA GP78 has performed to assess the viral load.

**Results -** To analyze any possible changes in the viral load due to any treatment condition the assay was performed. (A) Mean fold change of viral RNA expression post mimic transfection, SCFA treatment and JEV infection. (B) Mean fold change of viral RNA expression post inhibitor transfection, SCFA treatment and JEV infection. No significant change in the viral RNS levels were observed. Data is represented as mean fold change ± SEM. One-Way ANOVA followed by Tukey’s post hoc test was applied, p<0.05 was considered significance.


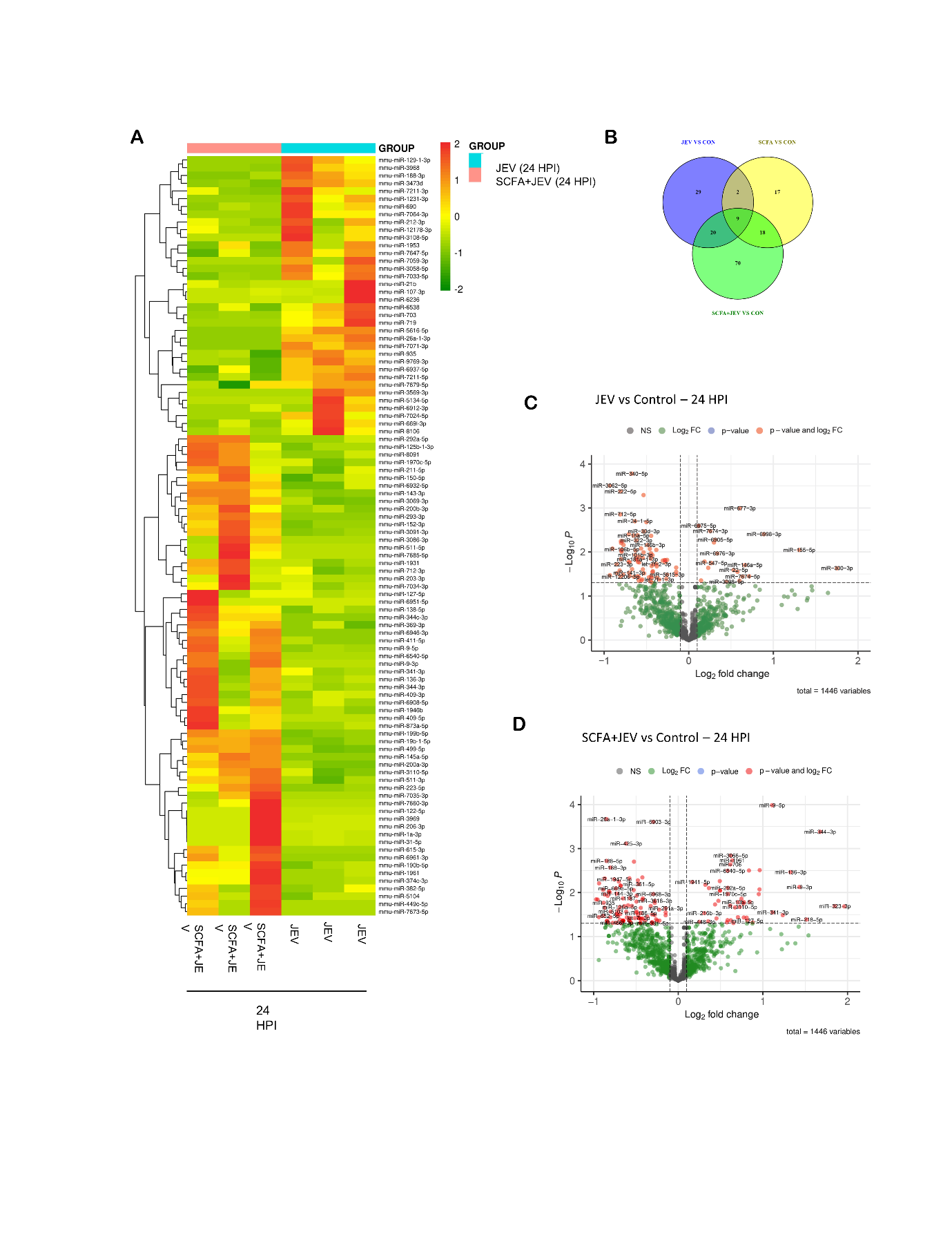


**Figure S5. Differential expression analysis of microRNAs post SCFA pre-treatment and/or JEV infection 24 HPI** Small RNA sequencing was performed post SCFA pre-treatment and JEV infection at 3 MOI (A) represents heat map depicting differentially expressed microRNAs in JEV group and SCFA+JEV group 24 HPI (B) Venn diagram showing the number of miRNAs differentially expressed across different conditions post 24 hours of infection (C) Volcano plot showing the differentially expressed miRNAs in JEV group versus control group (D) Volcano plot showing the differentially expressed miRNAs in SCFA+JEV group versus control group


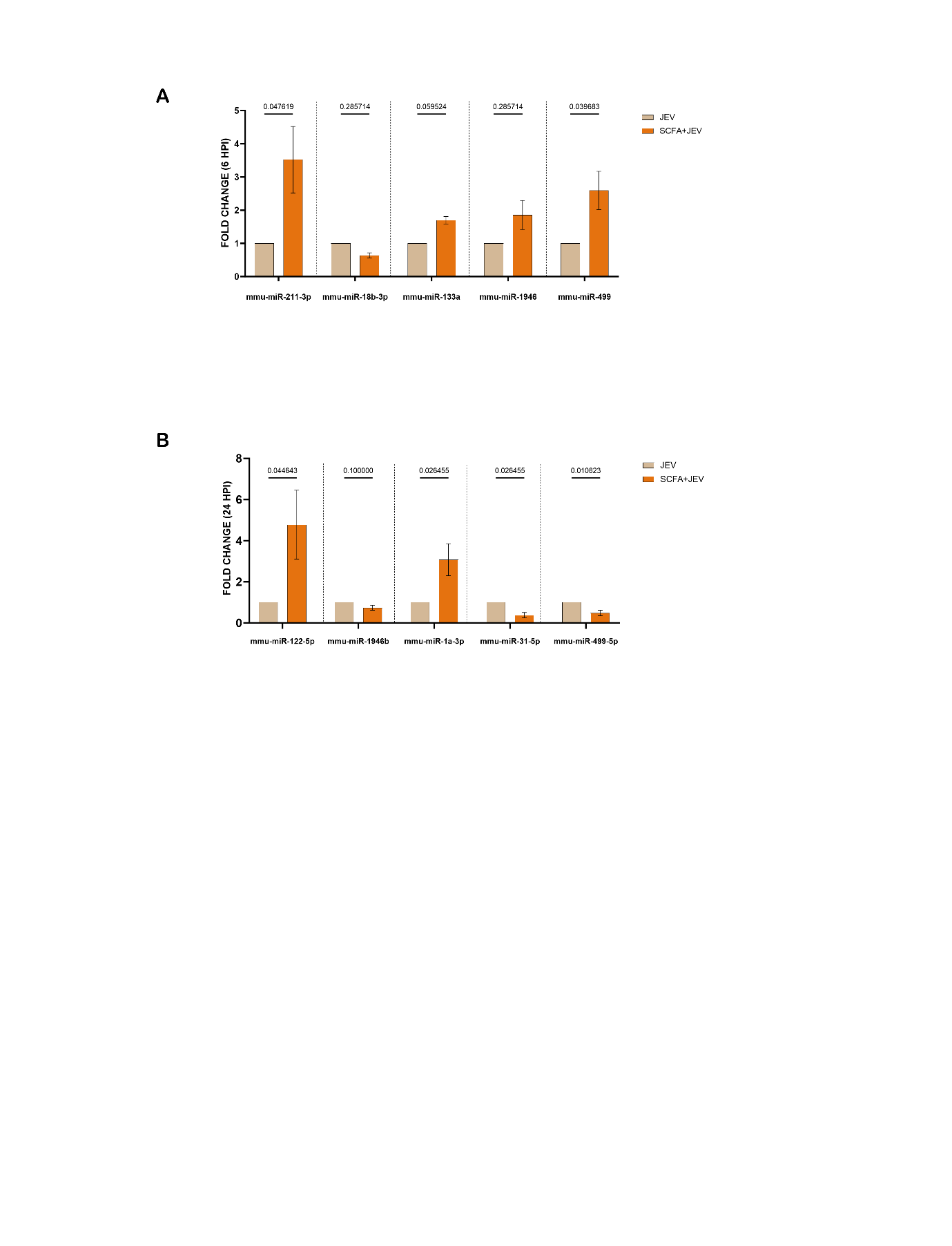


**Figure S6 Quantitative real time PCR validations of small RNA sequencing** SYBR green based real time PCR analysis was performed against selected miRNAs to validate the sequencing analysis. The figure depicts bar graphs showing the fold change of selected miRNA expression with respect to control (A) 6 HPI (B)24 HPI. Data are represented as mean fold change ± SEM. Mann-Whitney U test was performed with a minimum of 3 independent experiments to test for significance. P<0.05 was considered significant


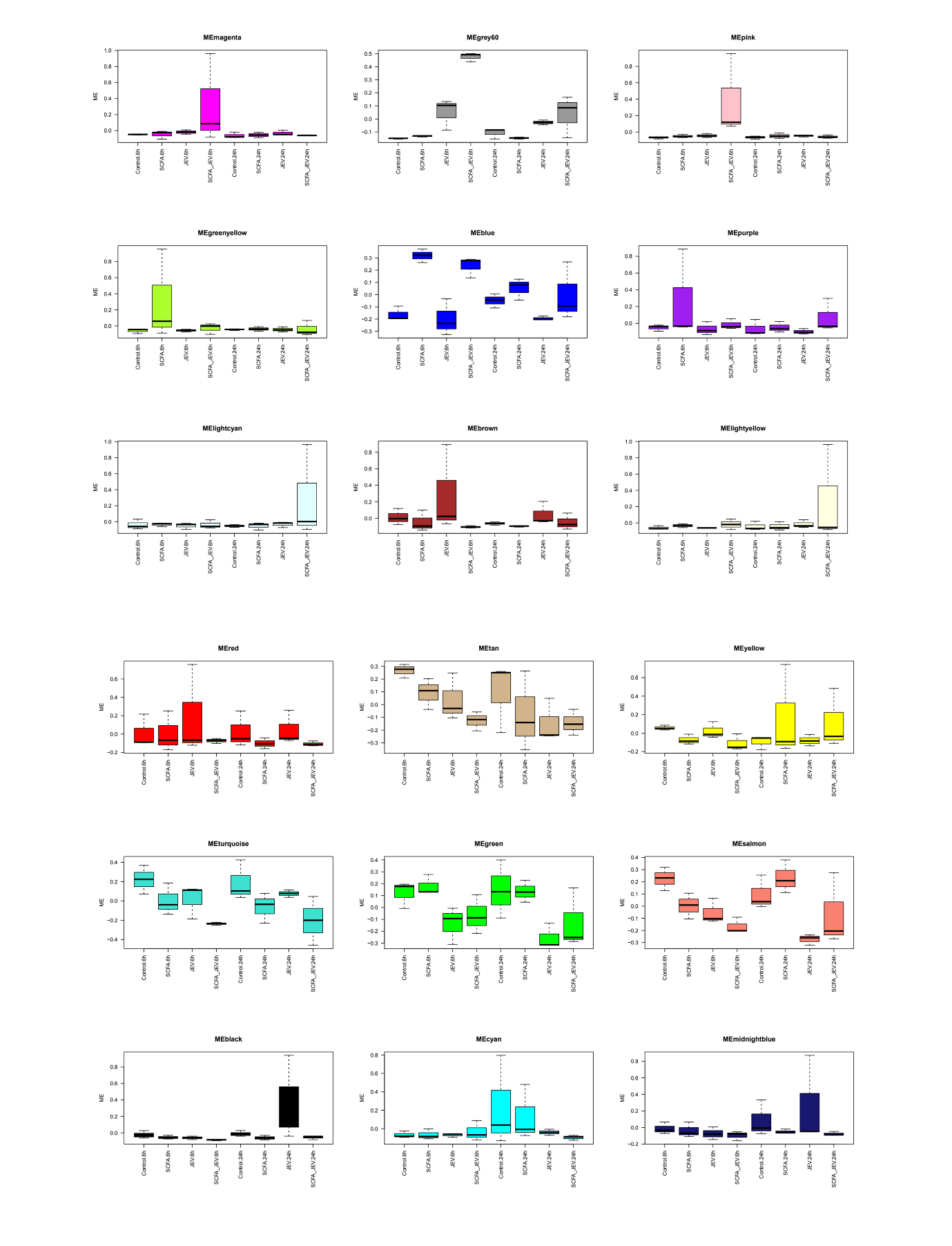


**Figure S7 WGCNA analysis derived microRNA co-expression modules.** 18 distinct miRNA modules were detected based on their differential expression in all four conditions – Control, SCFA, JEV and SCFA JEV across two time points. Each module was assigned a color, with the box plots representing the differential co-expression of the particular miRNA module across the given conditions.


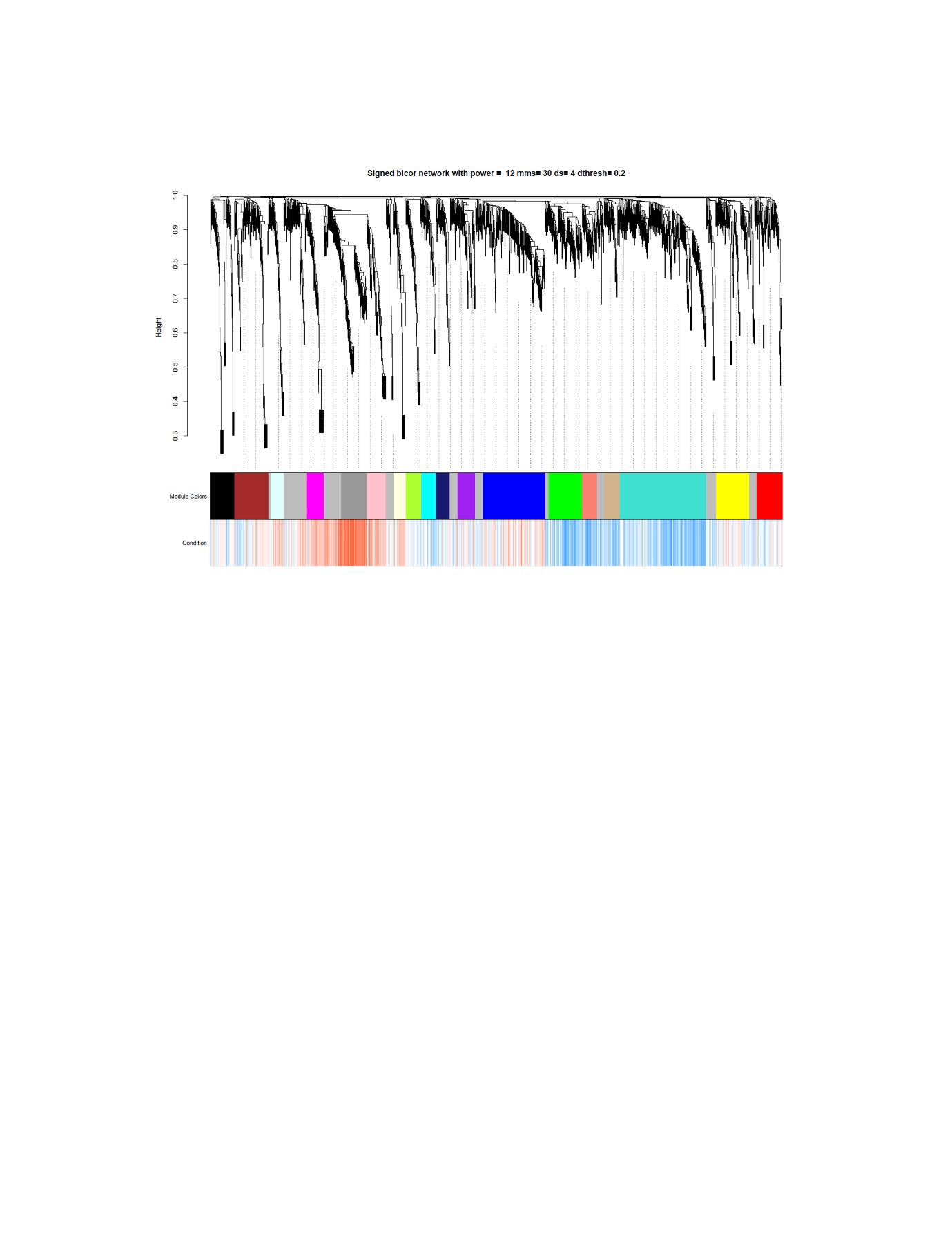


# Figure S8. WGCNA analysis based dendogram. Gene clustering tree derived by hierarchical clustering of adjacency-based dissimilarity to detect 18 co-expression microRNA clusters with corresponding color designations. Each color represents a module and the gray module indicates none co-expression among the microRNAs


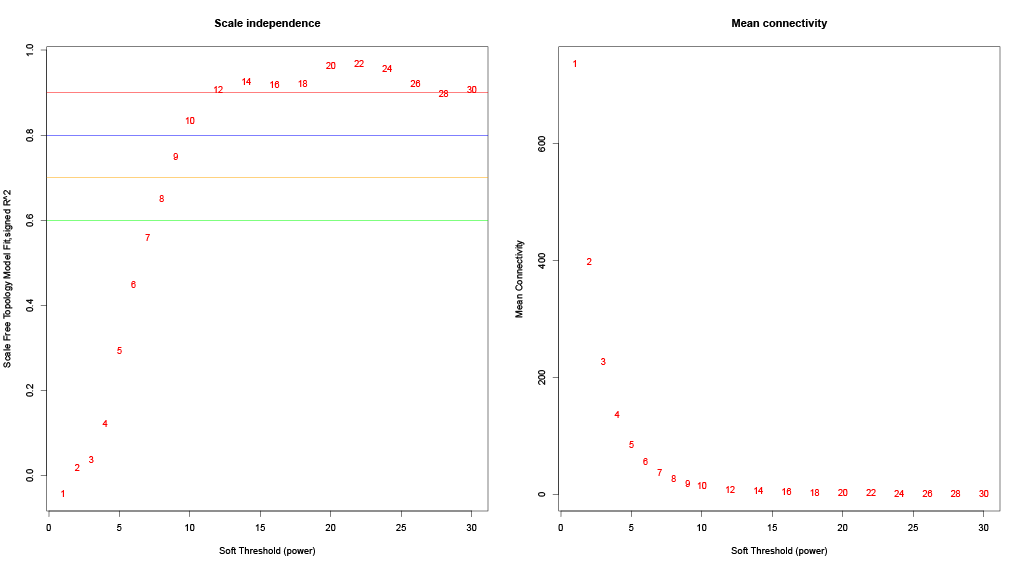


**Figure S9. Soft-power thresholding plot.**  the plot on the left shows scale-free topology fit index (x-axis) for different thresholding powers (x-axis). The plot on the right represents analysis of the main connectivity (degree, y-axis) for various thresholding powers (x-axis)

**
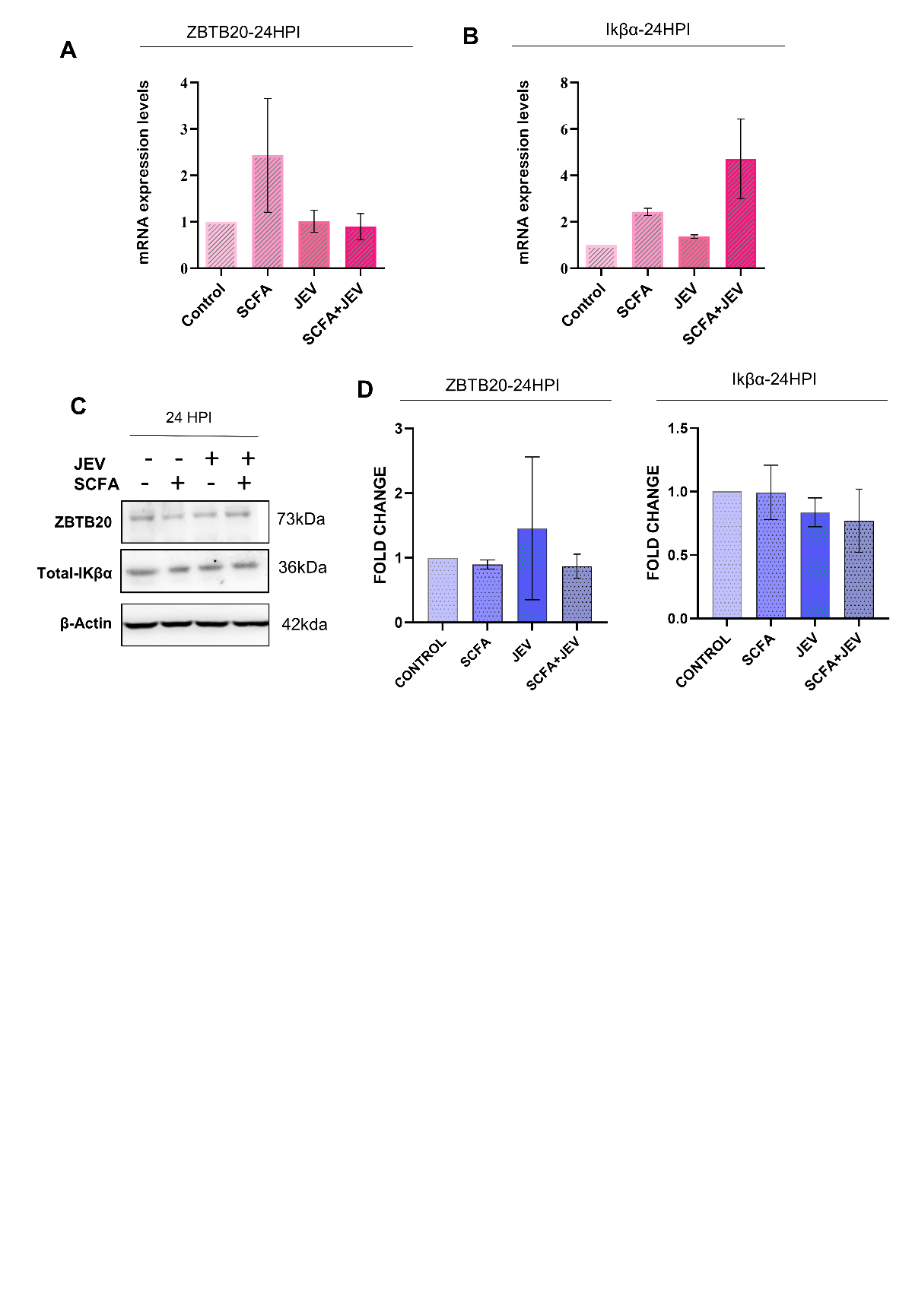
**

**Figure S10 Post-transcriptional regulation of ZBTB20 leads to transcriptional regulation of Iκβα gene. (**A) Bar graph representing mRNA expression levels of ZBTB20 gene across four conditions at 24HPI. (B) Bar graph representing mRNA expression level of Iκβα across four conditions at 24HPI. (C) representable immunoblots of ZBTB20 and Ikβα protein from whole cell lysate after desired treatment paradigm using SCFA and/or JEV (3MOI) 24HPI. Bar graphs showing the densitometric quantification of the immunoblots from panel. Data represented as mean fold change ± SEM with respect to control from a minimum of 3 independent experiments. P values were determined (*, P<0.05;**, P < 0.01;***, P < 0.001) using one way ANOVA, followed by Tukey’s post hoc correction.
